# An efficient multi-objective parrot optimizer for global and engineering optimization problems

**DOI:** 10.1038/s41598-025-88740-8

**Published:** 2025-02-11

**Authors:** Mohammed R. Saad, Marwa M. Emam, Essam H. Houssein

**Affiliations:** 1 Faculty of Computers and Information, Luxor University, Luxor, Egypt; 2 Faculty of Computers and Information, Minia University, Minia, Egypt; 3 Minia National University, Minia, Egypt

**Keywords:** Parrot optimizer, Multi-objective parrot optimizer, Pareto optimal solutions, Multi-objective optimization techniques, Computational science, Computer science

## Abstract

The Parrot Optimizer (PO) has recently emerged as a powerful algorithm for single-objective optimization, known for its strong global search capabilities. This study extends PO into the Multi-Objective Parrot Optimizer (MOPO), tailored for multi-objective optimization (MOO) problems. MOPO integrates an outward archive to preserve Pareto optimal solutions, inspired by the search behavior of Pyrrhura Molinae parrots. Its performance is validated on the Congress on Evolutionary Computation 2020 (CEC’2020) multi-objective benchmark suite. Additionally, extensive testing on four constrained engineering design challenges and eight popular confined and unconstrained test cases proves MOPO’s superiority. Moreover, the real-world multi-objective optimization of helical coil springs for automotive applications is conducted to depict the reliability of the proposed MOPO in solving practical problems. Comparative analysis was performed with seven recently published, state-of-the-art algorithms chosen for their proven effectiveness and representation of the current research landscape-Improved Multi-Objective Manta-Ray Foraging Optimization (IMOMRFO), Multi-Objective Gorilla Troops Optimizer (MOGTO), Multi-Objective Grey Wolf Optimizer (MOGWO), Multi-Objective Whale Optimization Algorithm (MOWOA), Multi-Objective Slime Mold Algorithm (MOSMA), Multi-Objective Particle Swarm Optimization (MOPSO), and Non-Dominated Sorting Genetic Algorithm II (NSGA-II). The results indicate that MOPO consistently outperforms these algorithms across several key metrics, including Pareto Set Proximity (PSP), Inverted Generational Distance in Decision Space (IGDX), Hypervolume (HV), Generational Distance (GD), spacing, and maximum spread, confirming its potential as a robust method for addressing complex MOO problems.

## Introduction

Design issues are fundamentally optimization problems requiring appropriate optimization methods and algorithms. The increasing complexity of modern design challenges has rendered traditional optimization methods, rooted in mathematical concepts, insufficient for providing timely and effective solutions. One such conventional method is the gradient-based algorithm, which tackles optimization problems using the gradient of the objective function^1^. In recent decades, addressing the limitations of standard optimization algorithms and developing new, efficient optimization algorithms has garnered significant attention. Technological advancements have spurred the creation of new optimization algorithms that offer high efficiency, accuracy, and speed in handling complex optimization problems^2^. Other significant challenges, including non-convexity, non-smoothness, and local optimization of search spaces, have primarily driven this research.

Due to these constraints, academics and experts have been inspired to develop novel metaheuristic algorithms to meet a range of optimization problems^3^. As information technologies evolve, numerous optimization problems emerge across diverse fields, such as engineering, bioinformatics, and geophysics^4,5^. Different optimization problems are classified as NP-hard, indicating that unless $NP = P$, they cannot be determined in a polynomial amount of time^6^. Consequently, precise mathematical methods are feasible only for small-scale issues.

Researchers have thus turned to approximation methods to find workable solutions within a reasonable time. These techniques are classified into heuristics and metaheuristics^7^. Heuristics are problem-specific, working well for particular problems but often failing in different contexts. In contrast, black-box optimizers, or general algorithmic frameworks, like metaheuristics, can solve almost any optimization problem^8^. Metaheuristics are a class of higher-level heuristics used to solve many optimization issues. Recently, many MH algorithms have been successfully applied to tackle complex problems^9,10^. The strength of these algorithms is their capacity to solve optimization problems of any size or complexity with a reasonable degree of quality. Numerous optimization issues, including continuous, discrete, single- and multi-objective, have been resolved using MH algorithms^11^.

MH algorithms leverage robust stochastic techniques and have gained significant recognition for their adaptability and performance in addressing complex challenges across various domains. Applications include global optimization problems^12^, production scheduling^13^, feature selection^14,15^, flex-route transit services^16^, wind energy conversion systems^17^, image segmentation^18^, and deep learning-based optimization^10,19^. A prominent category of metaheuristics is swarm intelligence, inspired by the collective behavior of animals, insects, and birds. Notable examples include liver cancer algorithm^20^, parrot optimizer^21^, slime mould algorithm^22^, Potter Optimization Algorithm (POA)^23^, Carpet Weaver Optimization (CWO)^24^, Fossa Optimization Algorithm (FOA)^25^, Addax Optimization Algorithm (AOA)^26^, and Dollmaker Optimization Algorithm (DOA)^27^. Moreover, there are some excellent algorithms developed, such as Sculptor Optimization Algorithm (SOA)^28^, Sales Training Based Optimization (STBO)^29^, Orangutan Optimization Algorithm (OOA)^30^, Tailor Optimization Algorithm (TOA)^31^, and Spider-Tailed Horned Viper Optimization (STHVO)^32^

Real-world issues frequently have several, sometimes opposing, goals. Consequently, single-objective optimization is not as appropriate for these real-world situations as multi-objective optimization problems (MOPs). To solve MOPs, each goal is usually approached as a single-objective OP and solved in order of decreasing significance^33^. As an alternative to increase overall performance, all single solvers can exchange information between iterations. In MOO, two or more competing objectives are concurrently optimized while subjecting constraints to construct Pareto-front optimal trade-off solutions. Different MOPs are optimized in numerous fields using MOO algorithms^34,35^.

Three general categories can be used to group MOO techniques: a priori, a posteriori, and interactive techniques^36^. Before the real optimization process starts, a priori approach, which relies on prior knowledge of the problem and the objectives, tries to uncover Pareto optimal solutions. One popular technique in these approaches is to take a multi-objective problem (MOP) and turn it into a single-objective problem by determining a single-objective function as the weighted sum of the normalized costs for each objective. Even if they are simple, they must be discussed with the decision-maker to establish the desired weights^37^.

After optimization, a Posteriori approach uses the solutions found throughout the process to determine Pareto optimal solutions. By keeping the MOP formulated, these approaches eliminate the requirement to combine various objectives or establish a set of weights. A posteriori techniques encompass a variety of evolutionary algorithms, such as simulated annealing, genetic algorithms, particle swarm optimization, NSGA-II, SPEA2, MOEA/D, and NSGA-III. These algorithms can identify Pareto optimal solutions in a single run and then use decision-making^38^.

Human intervention occurs during the optimization process when using interactive approaches. By using these techniques, the decision-maker can communicate with the optimization method and offer input on the answers the algorithm has produced. Interactive particle swarm optimization, interactive evolutionary strategies, and interactive genetic algorithms are examples of interactive techniques^39^.

Multi-objective evolutionary algorithms (MOEAs) have been gaining popularity and are widely adopted for addressing MOPs because of their efficiency in producing multiple Pareto optimal solutions in a single execution. This effectiveness results from using a population-based search method with few distinct dominance relationships. When used to simultaneously construct a robust approximation to the Pareto front (PF) and Pareto set (PS), MOEAs are very effective as an a posteriori approach^40^. Most existing MOEAs can be organized into four classes depending on their preference techniques: domination-based, indicator-based, decomposition-based, and hybrid MOEAs. MOEAs typically evaluate the function values of all newly generated solutions to assess the quality of the selection process. As such, before these algorithms reach convergence, they must frequently undergo multiple function evaluations^41^.

However, traditional optimization strategies often fall short of delivering satisfactory long-term results. These conventional techniques face several limitations, including sensitivity to initial estimates, reliance on the accuracy of differential equation solvers, and a high probability of getting stuck in the local region^42^. Various advanced algorithms have been developed to address these complex optimization issues efficiently.

The PO, developed by T. Junbo Lian et al.^43^, is a strong algorithm successfully used for various engineering issues, manufacturing methods, and scientific models. Building on the strengths of PO, we were inspired to create a multi-objective version, termed MOPO, specifically designed to tackle MOO problems. The effectiveness of MOPO is rigorously evaluated by employing the CEC’2020 benchmarks. Also, MOPO is compared against seven well-established optimization algorithms: MOPSO^44^, NSGA-II^45^, MOGWO^46^, MOWOA^47^, MOSMA^48^, IMOMRFO^49^, and MOGTO^50^. The reasons behind selecting this comparison pool are that it includes some state-of-the-art algorithms in the domain of MOO, such as MOPSO and NSGA-II; it also includes very recent and strong algorithms, such as MOSMA, IMOMRFO, and MOGTO. The main reason is it employs the same Pareto-based archiving mechanisms using the non-dominated ranking and crowding distance tactics that are incorporated with the proposed MOPO for a fair comparison. Generally, comparisons should be made with state-of-the-art, recent, and strong algorithms. The Proposed MOPO can hold onto the finest candidates among the Pareto optimum solutions. With an additional crowding distance (CD) operator to enhance the diversity and coverage of the premium solutions, the individuals in the swarm update their positions in a manner akin to the original PO. Furthermore, the parrot’s premium host places come from the archive of non-dominated solutions (NDSs), guaranteeing thorough investigation and exploitation throughout the optimization procedure.

To summarize, this paper has made the following contributions:

- The notable advantages of the PO inspired the development of its multi-objective version, MOPO, and its performance is benchmarked against seven cutting-edge MOO algorithms.
- The CEC’2020 benchmarks are used to assess the efficacy of MOPO.
- To demonstrate the robustness of MOPO, the PSP, IGDX, and HV indicators are employed.
- MOPO’s performance is assessed using four specific engineering design challenges and eight popular constrained and unconstrained test cases. Evaluation metrics include GD, spacing, and maximum spread.
- Moreover, the real-world MOO of helical coil springs for automotive applications is addressed to depict the reliability of the proposed MOPO to solve real-world challenges.
- The Wilcoxon test, Friedman test, and various performance metrics are utilized to estimate the significance of MOPO compared to seven well-established optimization algorithms.

The remainder of the paper is arranged as follows. “Related Work” Section includes an overview of the previous studies. In “Basic Concepts of Multi-Objective Optimization Problems (MOPs)” Section, the basic ideas of MOP are explained. “The Parrot Optimizer (PO)” Section contains the original PO. In “The Proposed Multi-Objective Parrot Optimizer (MOPO)” Section, the suggested MOPO is presented. “Excremental Results Analysis and Discussion” Section analyzes the experimental results. “Limitations and Advantages of the Proposed MOPO” Section presented the advantages and limitations of the proposed method. “Conclusion and Future Directions” Section illustrates the conclusions along with some suggestions for further investigation.

## Related work

MOO is a crucial field of study in optimization, addressing issues where several objective functions must be simultaneously optimized. Unlike single-objective optimization, Pareto-optimal solutions, or a range of equally viable alternatives, are produced by MOO^51^. These solutions provide a trade-off with the different objectives, making MOO particularly relevant in real-world applications where multiple criteria must be considered. The field of MOO has evolved significantly since its inception. Early approaches primarily aggregated multiple objectives into a single composite objective function. However, these methods often fail to capture the complexity of trade-offs between different objectives. Introducing Pareto-based methods marked a significant milestone, allowing for a more nuanced approach to handling multiple objectives. Several algorithms have been developed to address MOO problems, each with its advantages and limitations:

*Genetic Algorithms (GAs):* One of the earliest and most widely used approaches is the Non-dominated Sorting Genetic Algorithm (NSGA) and its enhanced variant NSGA-II^45^. NSGA-II is renowned for its efficient, non-dominated sorting procedure and ability to maintain diverse solutions. This method starts by employing a non-dominated sorting mechanism to label Pareto sets, starting from the first non-dominated front. The selection procedure is then guided by the CD metric that a crowded-comparison operator assigns to each solution. The system optimizes solutions in less populated locations and emphasizes elitist alternatives with lower domination ratings for survival, hence maintaining diversity. To ensure the population size remains consistent with the initial size, the selection of non-dominated individuals is repeated multiple times. These steps continue until a predefined termination condition is met.

*Particle Swarm Optimization (PSO):* MOPSO adapts the principles of particle swarm optimization to handle multiple objectives. MOPSO has shown promising results in maintaining diversity and convergence^52^. Coello and Lechuga^53^ introduced the MOPSO algorithm, demonstrating a more time-efficient performance than NSGA-II. MOPSO uses a fixed-capacity repository to hold NDS that can be used in later stages. The algorithm partitions the search space into equal-sized hypercubes to find less-explored regions within the objective function’s search space. This methodology guarantees a wide distribution of solutions throughout the PF and contributes to preserving solution variety. Consequently, this gives designers many options rather than concentrating solutions in specific regions.

*Differential Evolution (DE):* Multi-objective Differential Evolution (MODE) is a widely recognized technique celebrated for its robustness and simplicity. MODE algorithms have effectively tackled various complex optimization problems^54^. In a comprehensive study by Gunantara^55^, the application of evolutionary MOO algorithms to engineering problems is thoroughly examined. Various MOO techniques have been created to optimize MOPs, including the multi-objective NSGA-III^56^, MODE^57^, MOGWO^46^, Dragonfly algorithm^58^, MO self-adaptive multi-population based Jaya algorithm (SAMP-Jaya)^59^, and MO moth flame optimization^60^. These studies highlight the efficacy of meta-heuristic algorithms in optimizing MOPs and comparing the true Pareto optimal front for various complex problems.

Numerous comparative studies have evaluated the performance of different MOO algorithms across various benchmark problems. These studies provide valuable wisdom into the powers and shortcomings of each approach. For instance, a MOO algorithm named MOCGO/DR based on Decomposition (MOCGO/D) was suggested by Yacoubi et al.^41^. This algorithm divides the MOP into single-objective sub-problems during decomposition using a Normalized Boundary Intersection (NBI) technique. Furthermore, the Multi-Objective Exponential Distribution Optimizer (MOEDO), which integrates elite non-dominated sorting and CD approaches, was proposed by Kalita et al.^11^. MOEDO incorporates an information feedback mechanism (IFM) to balance exploration and exploitation. This enhances convergence and lessens the possibility of stalling in local optima, a common drawback in conventional optimization techniques. Zitzler et al.^61^ introduced the strength Pareto evolutionary algorithm (SPEA). Knowles and Corne^62^ created the Pareto archived evolution strategy (PAES-2). Tejani et al.^63^ presented enhanced multi-objective symbiotic organisms search (MOSOS) designed to solve engineering optimization issues.

Furthermore, A new multi-objective version of the Geometric Mean Optimizer (GMO), called MOGMO, was presented by Pandya et al.^64^. MOGMO adopts a robust offspring production and selection process, combining the standard GMO with an elite, non-dominated sorting mechanism to locate Pareto optimal solutions successfully. A grid-based BFO method with multiple resolutions for MOPs was proposed by Junzhong Ji et al.^65^ (MRBFO). Four optimization mechanisms-chemotaxis, conjugation, reproduction, and elimination and dispersal-are redesigned by MRBFO. Moreover, Houssein et al.^66^ introduced a multi-objective algorithm of the self-adaptive Equilibrium Optimizer (self-EO), MO-self-EO. The MO-self-EO was validated on two categories of multi-objective problems: the CEC’2020 MO routines and MO engineering design challenges. The algorithm employs a selection strategy to enhance convergence and solution diversity, combining CD with an IFM. Meanwhile, Dhiman et al.^67^ suggested the Multi-Objective Spotted Hyena Optimizer (MOSHO), featuring a fixed-size archive to manage NDS. Additionally, Zarbakht et al.^68^ presented the Multi-Layer Ant Colony Optimization (MLACO) algorithm for solving multi-objective community detection problems. Using a parallel search method, MLACO improves convergence and demonstrates computational efficiency.

Moreover, Seyedali et al.^9^ suggested an Ant Lion Optimizer (ALO) multi-objective version to tackle engineering design issues. The Multi-Objective Seagull Optimization Algorithm (MOSOA) was introduced by Dhiman Gaurav et al.^69^ to solve various optimization problems. This was more recently. Shankar et al.^70^ created an enhanced multi-objective method to improve solution spread and convergence in engineering design challenges. Mozaffari et al.^71^ presented the Synchronous Self-Learning Pareto Strategy (SSLPS), a multi-objective algorithm for vector optimization. Also, Kumar et al.^72^ suggested the Multi-Objective Thermal Exchange Optimization (MOTEO) method for truss design. Based on Newton’s law of cooling, this multi-objective version enhances the single-objective TEO algorithm, integrating non-dominated sorting and CD methods for improved performance.

Furthermore, a variant of the gorilla troops optimizer (GTO) was developed in^50^ to tackle issues with MO optimization. It refers to this version as MOGTO. Houssien et al.^73^ proposed a new approach by integrating a multi-objective technique with the bird swarm algorithm (BSA), resulting in the development of the MBSA method. The MBSA successfully generates NDS while maintaining diversity among the optimal solutions. An MO form of advanced moth flame optimization (MFO) based on CD and NDS was presented by Saroj et al.^74^. To address the shortcomings of MFO, a weight strategy (WS) and a mathematical quasi-reflection-based learning (QRL) method were first introduced to the traditional MFO. Subsequently, this advanced MFO has been expanded into a MO variation called MOQRMFO. The MnMOMFO, a novel NDS and CD-based MO variation of the MFO method for MO optimization issues, was introduced in^75^. The method addresses the shortcomings of MFO and enhances its performance by incorporating notions from arithmetic and geometric means. Subsequently, this improved MFO was configured into an MO version, and NDS and CD techniques were utilized to attain a Pareto optimal front that was uniformly distributed.

Recent advancements in MOO have increasingly focused on hybrid algorithms, combining various optimization techniques’ strengths. Hybrid multi-objective evolutionary algorithms (MOEAs) have become particularly popular for addressing complex MOPs, leveraging the combined strengths of multiple algorithms^76^. The two main things to think about with these hybrid algorithms are choosing which algorithms to combine and how to integrate them. Recent work has combined several tactics to produce progeny populations^77^. However, further exploration is needed in other domains. Notable examples of hybrid MOEAs include the Hybrid Multi-Objective Evolutionary Algorithm based on the Search Manager Framework for big data optimization problems^76^, the Hybrid Selection Multi/Many-Objective Evolutionary Algorithm^77^, the Hybrid TOPSIS-PR-GWO Approach for multi-objective process parameter optimization^78^, and the Simulated Annealing Based Undersampling (SAUS) method for tackling class imbalance in multi-objective optimization^79^. Additionally, Moghdani et al.^80^ presented the MO volleyball premier league technique (MOVPL) to address global optimization problems with many target jobs. Teams vying for spots in a top volleyball league served as the inspiration for this optimization approach.

Other widely recognized MOO algorithms include the Multi-Objective Equilibrium Optimizer (MOEO)^81^, the MOSMA^82^, the Multi-Objective Arithmetic Optimization Algorithm (MOAOA)^83^, and the multi-objective Coronavirus disease optimization algorithm (MOCOVIDOA)^37^. Additionally, the Multi-Objective Evolutionary Algorithm based on Decomposition (MOEA/D)^84^, and the Multi-Objective Multi-Verse Optimization (MOMVO)^85^.

The Non-dominated Sorting Grey Wolf Optimizer (NS-GWO)^86^, the Multi-Objective Gradient-Based Optimizer (MOGBO)^87^, the Multi-Objective Plasma Generation Optimizer (MOPGO)^88^, the Multi-Objective Marine Predator algorithm for solving Multi-Objective Optimization problems (MOMPA)^89^, and the Non-dominated Sorting Harris Hawks Optimization (NSHHO)^90^ are some more significant contributions. The Decomposition-Based Multi-Objective Symbiotic Organism Search (MOSOS/D)^91^ and the Decomposition-Based Multi-Objective Heat Transfer Search (MOHTS/D)^92^ are two other noteworthy algorithms.

In the context of the recent related studies, the work in^93^ proposed a dynamic holographic modeling approach for the augmented visualization of digital twin scenarios for bridge construction. A dynamic segmentation technique with variable screen size was developed to generate holographic scenes more effectively, and a motion blur control approach was developed to increase holographic scene rendering efficiency based on human visual features. Finally, the prototype system was created, and the necessary experimental analysis was accomplished. Design for remanufacturing (DfRem) is an essential concept that tries to limit carbon emissions during the remanufacturing phase from the start. However, low-carbon standards and customer preferences are not fully considered when developing schemes, which may limit the potential to cut carbon emissions. To solve the issue, the study in^94^ developed a DfRem scheme generation approach that combines the quality function deployment for low-carbon (QFDC) model with the functional-motion-action-structure (FMAS) model. Furthermore, the work in^95^ also suggested a multihead attention self-supervised (MAS) representation model, which is a self-supervised learning-based sensor feature extraction network. Similarly, the work described in^96^ presented causal intervention visual navigation (CIVN), which is based on deep reinforcement learning (DRL) and causal intervention. Appliance types and power consumption patterns differ significantly across industries. This can lead to inconsistencies in the identification results of traditional appliance load monitoring methods across industries. For that, a non-intrusive appliance load monitoring (NIALM) solution for diverse industries based on multiscale spatio-temporal feature fusion has been proposed in^97^. The efficient tracking and capture of escaping targets using unmanned aerial vehicles (UAVs) is the focus of the UAV pursuit-evasion problem. This topic is crucial for public safety applications, especially when intrusion monitoring and interception are involved. The study in^98^ proposed a novel swarm intelligence-based UAV pursuit-evasion control framework, called the “Boids Model-based DRL Approach for Pursuit and Escape” (Boids-PE), which combines the advantages of swarm intelligence from bio-inspired algorithms and deep reinforcement learning (DRL) in order to address the difficulties of data acquisition, real-world deployment, and the limited intelligence of existing algorithms in UAV pursuit-evasion tasks. Moreover, a collaborative imaging technique using a reverse-time migration (RTM) algorithm and a zero-lag cross-correlation imaging condition was presented in^99^. It was developed independently from ground-penetrating radar (GPR) and pipe-penetrating radar (PPR) data. Likewise, in order to improve the degree of fairness while meeting the required priority requirements, the study in^100^ examined and defined the slice admission control (SAC) problem in 5G/B5G networks as a nonlinear and nonconvex MOO problem. Consequently, to address the issue, the study also suggested a heuristic approach known as prioritized slice admission control considering fairness (PSACCF).

Furthermore, recent studies have been introduced in the domain of optimization, such as in^101^, which suggested a novel solution for nonconvex problems involving multiple variables. This is particularly true for problems that are usually resolved by an alternating minimization (AM) strategy, which divides the original optimization problem into a number of subproblems that correspond to each variable and then iteratively optimizes each subproblem using a fixed updating rule. In^102^, a strategy for simultaneously optimizing mobile robot base position and cabin angle using a homogeneous stiffness domain (HSD) index for large spaceship cabins was presented. The work in^103^ looked at the joint design of a multiple-input multiple-output (MIMO) broadcast waveform and a receive filter bank for RSU-mounted radar in a spectrally packed vehicle-to-infrastructure communication environment. A non-convex problem was presented with the criterion of maximizing the average signal-to-interference-plus-noise ratio (SINR), which involves the weighted-sum waveform energy over the overlaid space-frequency bands, as well as energy and similarity constraints. In this work, an iterative approach was proposed for solving the joint optimization issue. Similarly, the study in^104^ developed a thermal-elastohydrodynamic mixed lubrication model for journal-thrust linked bearings that takes into account pressure, flow, and thermal continuity conditions and validated it with trials. Based on this, preliminary research was conducted into the effect of textural characteristics on lubrication under real-world heavy load situations. The orthogonal test design was then utilized to identify the texture parameter with the highest influence on optimization. The PSO approach was then used for synchronous texture design for both the journal and the thrust sections. Additionally, a method for identifying uneven industrial loads using optimized CatBoost with entropy characteristics was developed^105^. The study in^106^ addressed the refined oil distribution problem with shortages through a multi-objective optimization strategy from the perspective of oil marketing company decision-makers. The modeling and solving approach included creating a crisp multi-depot vehicle routing model, developing a robust optimization model, and proposing the MOPSO model. Moreover, in^107^, a multi-objective optimization model for an adaptive maintenance window-based opportunistic maintenance (OM) method was proposed.

Despite significant progress, several challenges remain in the field of MOO. One major challenge is the scalability of algorithms for high-dimensional problems. Another issue is the need to handle constraints efficiently in MOO problems. Furthermore, developing algorithms that can dynamically adapt to changing problem landscapes is an ongoing area of research. In this work, we propose a novel multi-objective optimization algorithm, the MO Parrot Optimizer (MOPO), to address some of the existing gaps in the field. By leveraging innovative strategies inspired by natural phenomena, MOPO provides a reliable and effective method for resolving challenging MOO issues.

## Basic concepts of multi-objective optimization problems (MOPs)

Solving single-objective optimization problems is generally simpler than solving MOPs. This is mainly because only one distinct solution is subject to one objective function. In single-objective scenarios, the absolute optimal solution is found, and the singularity of the objective makes it easy to compare solutions. On the other hand, more solutions to MOO issues complicate evaluating the answers^108^.

The mathematical formula of a multi-objective optimization problem is presented as^109^:

1 $$\begin{aligned} & \text{ Minimization } \text{: } \quad F(\vec {x})=\left\{ f_{1}(\vec {x}), f_{2}(\vec {x}), \ldots , f_{obj}(\vec {x})\right\} \nonumber \\ & \quad \begin{array}{ll} \text{ subject } \text{ to: } & g_{i}(\vec {x}) \ge 0, \quad i=1,2, \ldots , Q\\ & H_{i}(\vec {x})=0, \quad i=1,2, \ldots , P \\ & L_{i} \le U_{i} \quad , i=1,2, \ldots , D \end{array} \end{aligned}$$

Here, the symbols $Q$, $P$, and $D$ stand for the number of variables, objective functions, equality constraints, and inequality constraints in that order. $L_i$ and $U_i$ denote the lower and upper bounds of the $i$-th parameter.

### Pareto dominance

Let $\vec {x}$ and $\vec {y}$ be two solutions with cost functions represented as:

2 $$\begin{aligned} \vec {x} = (x_1, x_2, \ldots , x_k) \quad \text {and} \quad \vec {y} = (y_1, y_2, \ldots , y_k) \end{aligned}$$

**Fig. 1:**
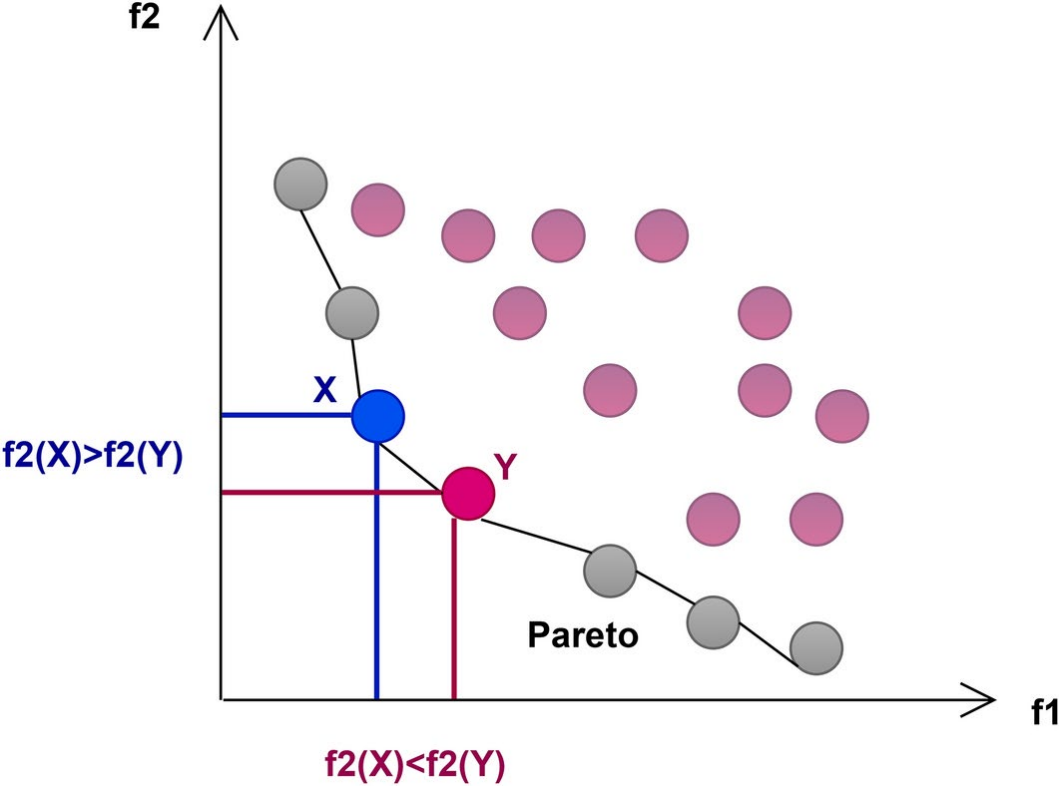
Pareto dominance.

For a minimization problem as shown in Fig. 1, solution $\vec {x}$ is expressed to overpower solution $\vec {y}$ (represented as $\vec {x} \preceq \vec {y}$) if none of $\vec {y}$’s cost elements are less than the equivalent cost elements of $\vec {x}$, and at least one element of $\vec {x}$ is smaller than that of $\vec {y}$. This can be formally defined as:

3 $$\begin{aligned} \forall i \in \{1, 2, \ldots , k\} : f_i(\vec {x}) \le f_i(\vec {y}) \quad \text {and} \quad \exists i \in \{1, 2, \ldots , k\} : f_i(\vec {x}) < f_i(\vec {y}) \end{aligned}$$

### Pareto optimality

The idea of Pareto optimality is dependent on Pareto dominance^110^:

A solution $\vec {x} \in X$ is called Pareto-optimal if and only if there is no $\vec {y} \in X$ such that $\vec {y} \preceq \vec {x}$.

### Pareto optimal set

The Pareto optimal set $P_s$ comprises all NDS to a specified problem. Mathematically, it is described as:

4 $$\begin{aligned} P_s := \{\vec {x} \in X \mid \not \exists \vec {y} \in X \text { such that } \vec {y} \preceq \vec {x}\} \end{aligned}$$

**Fig. 2:**
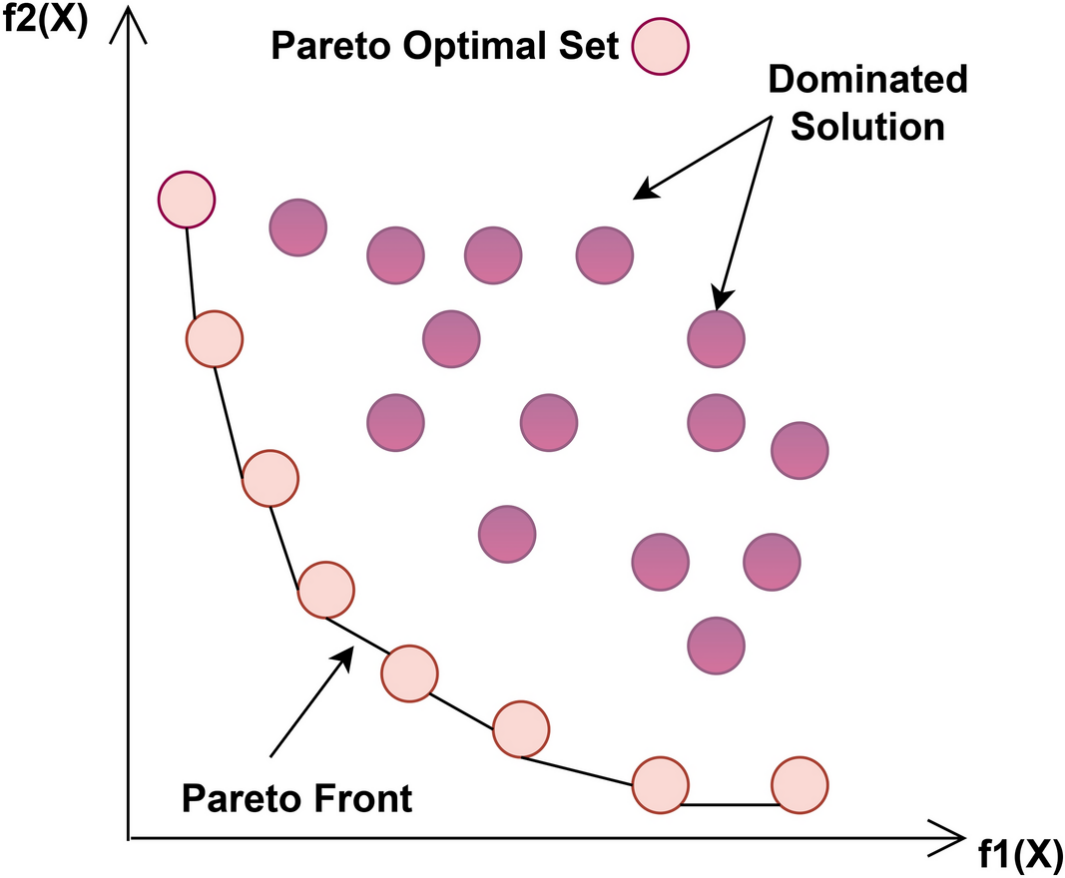
Pareto optimal solutions.

The Pareto optimal set is visually represented in Fig. 2, showing the ensemble of optimal solutions.

### Pareto optimal front

The Pareto optimal front $P_f$ denotes the set of Pareto-optimal solutions mapped in the objective area. It can be described as:

5 $$\begin{aligned} P_f = \{F(\vec {x}) \mid \vec {x} \in P_s\} \end{aligned}$$

As Fig 2 explains, the PF $P_f$ denotes. the optimal set in the objective area.

**Fig. 3:**
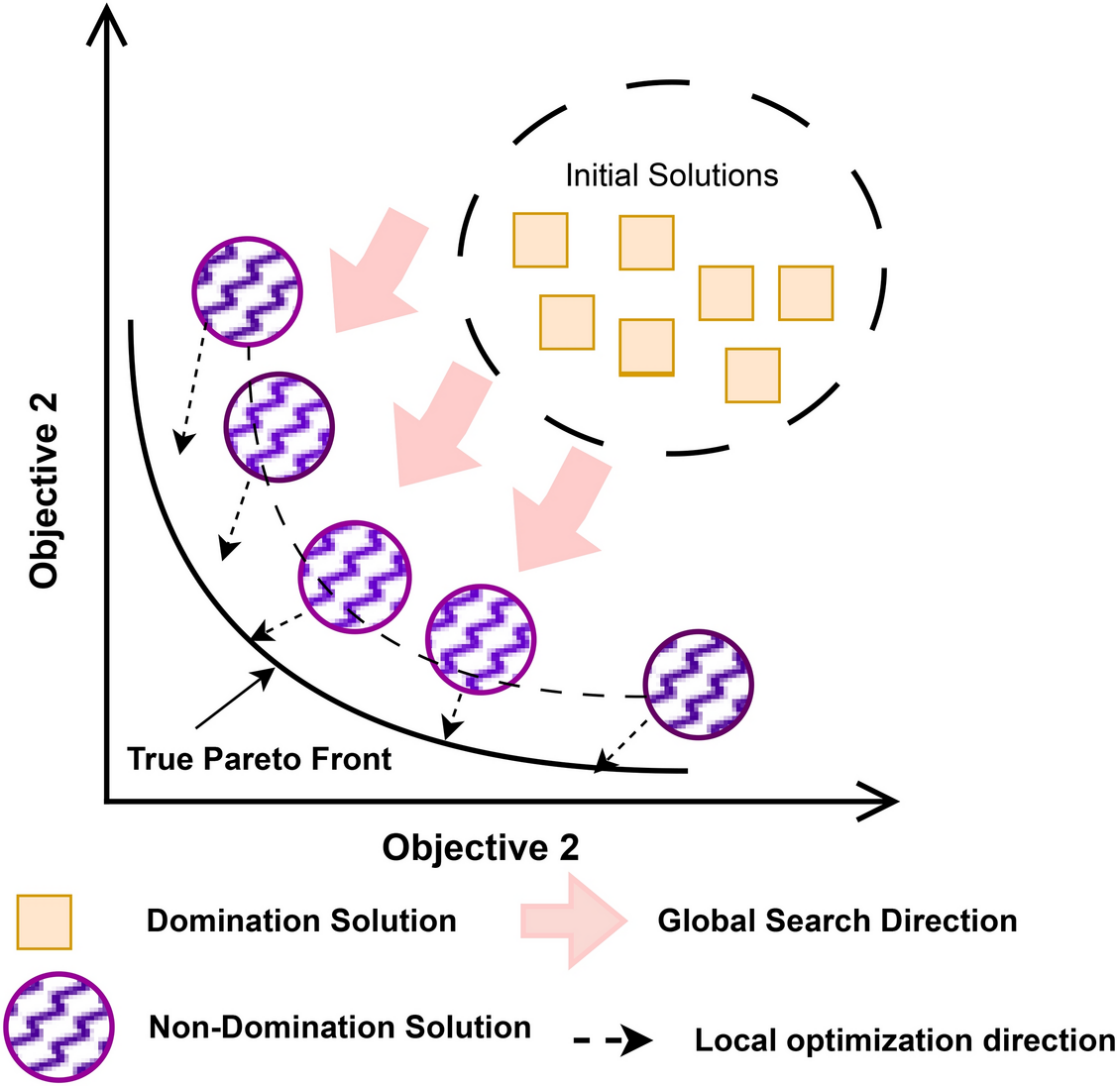
Multiobjective optimization process.

The MOO process is typically illustrated by an intermediate or current front of NDS discovered by the optimization process, which approximates the true PF, as depicted in Fig. 3.

NSGA-II is a multi-objective genetic algorithm that is frequently used^45^. In NSGA-II, Pareto sets are identified and labeled, beginning with the first non-dominated front and using a non-dominated sorting strategy.

## The parrot pptimizer (PO)

PO is a modern optimization algorithm motivated by the key behaviors of Pyrrhura Molinae parrots. This algorithm emulates four distinct behavioral traits observed in trained Pyrrhura Molinae parrots-namely, foraging, resting, communication, and fear of strangers-to perform the core optimization tasks of exploration (diversification) and exploitation (intensification). These behaviors are modeled mathematically to simulate the optimization process. The following outlines the mathematical framework of the PO:

### Foraging attitude

In the foraging attitude in PO, parrots mostly use observation to estimate the approximate food position or consider the owner’s position. Then, they fly in the direction of the estimated position. The positional movement, therefore, obeys the following Eq. (6):

6 $$\begin{aligned} C_i^{t+1}=\left( C_i^t-C_{\text{ optimal }}\right) * L(D)+\operatorname {rand}(0,1) * \left( 1-\frac{t}{\text{ Max}_{\text{ cy }}}\right) ^{\frac{2 t}{\text{ Max}_{\text{ cy }}}} * C_{\text{ mean } }^t \end{aligned}$$

where ${Max}_{\text{ cy }}$ indicates the maximum number of cycles and rand(0, 1) represents a random number in [0, 1].

In Eq. (6), $C_i^t$ denotes the current position, while $C_i^{t+1}$ represents the position after the next update. $C_{\text {mean}}^t$ is the average position of the current agents, and *L*(*D*) signifies the Levy distribution used to model the flight behavior of the parrots. $C_{\text {optimal}}$ refers to the best position discovered from initialization to the current iteration and is also known as the host position. The variable *t* represents the current cycle count. The term $\left( C_i^t - C_{\text {optimal}}\right) * L(D)$ captures the movement of the parrots relative to the host position, while $\operatorname {rand}(0,1) * \left( 1 - \frac{t}{\text {Max}{\text {cy}}}\right) ^{\frac{2t}{\text {Max}{\text {cy}}}} * C_{\text {mean}}^t$ describes the influence of the overall population position on the direction of movement towards food sources.

The average position of the current swarm, denoted as $C_{\text {mean}}^t$, is calculated using the formula provided in Eq. (7).

7 $$\begin{aligned} C_{\text{ mean }}^t=\frac{1}{S_{in}} \sum _{k=1}^{S_{in}} C_k^t \end{aligned}$$

where $S_{in}$ denotes the swarm size.

The Levy distribution can be derived using the rule specified in Eq. (8), with $\gamma$ set to 1.5.

8 $$\begin{aligned} \left\{ \begin{array}{l} L(D)=\frac{\mu \cdot \sigma }{|v|^{\frac{1}{\gamma }}} \\ \mu \sim N(0, \operatorname {D}) \\ v \sim N(0, \operatorname {D}) \\ \sigma =\left( \frac{\Gamma (1+\gamma ) \cdot \sin \left( \frac{\pi \gamma }{2}\right) }{\Gamma \left( \frac{1+\gamma }{2}\right) \cdot \gamma \cdot 2^{\frac{1+\gamma }{2}}}\right) ^{\gamma +1} \end{array}\right. \end{aligned}$$

### Remaining attitude

The major behavior of Pyrrhura Molinae, a very gregarious bird, is to take off suddenly and land on any area of its owner’s body, where it stays motionless for a while. This procedure can be expressed mathematically as:

9 $$\begin{aligned} C_i^{t+1}=C_i^t+C_{\text{ optimal }} * L(D) + rand(0,1) * ones(1, D) \end{aligned}$$

where *ones*(1, *D*) indicates the whole vector of dimension *D*. $C_{\text{ optimal }} * L(D)$ indicates the flight to the host, and $rand(0,1) * ones(1, \text{D})$ indicates the procedure of randomly stopping at a portion of the host’s body.

### Communicating attitude

Parrots of the Pyrrhura Molinae family are naturally sociable and exhibit close group communication. Both flying to the flock and communicating without flying to the flock are included in this communication attitude. Both behaviors are taken to occur equally likely in the PO, and the current population’s mean position represents the flock’s center. This procedure can be expressed mathematically as:

10 $$\begin{aligned} \begin{aligned}&C_i^{t+1} =&{\left\{ \begin{array}{ll} 0.2 * rand (0, 1) * \left( 1-\frac{t}{\text{ Max}_{\text{ cy }}}\right) * (C_i^{t} - C_{mean}^{t}), & Q \le 0.5 \\ 0.2 * rand (0, 1) * \exp \left( -\frac{t}{\operatorname {rand}(0, 1) * \text{ Max}_{\text{ cy }}}\right) , & Q > 0.5 \end{array}\right. } \end{aligned} \end{aligned}$$

where, $0.2 \cdot \operatorname {rand}(0, 1) \cdot \left( 1 - \frac{t}{\text {Max}_{\text {cy}}}\right) \cdot \left( C_i^t - C_{\text {mean}}^t\right)$ represents the operation of an individual entering a parrot’s group for communication, and $0.2 \cdot \operatorname {rand}(0, 1) \cdot \exp \left( -\frac{t}{\operatorname {rand}(0, 1) \cdot \text {Max}_{\text {cy}}}\right)$ represents the operation of an individual flying out directly after communication. Both conducts are implemented based on a randomly developed $Q$ in the range $[0,1]$.

### Fear of strangers attitude

Birds, particularly Pyrrhura Molinae parrots, have an innate fear of foreigners. The following mathematical illustration shows how they behaved, avoiding foreign people and hiding with their masters while they were looking for a haven Eq. (11):

11 $$\begin{aligned} \begin{aligned} C_i^{t+1}&=C_i^t+\operatorname {rand}(0,1) * \cos \left( 0.5 \pi * \frac{t}{\text{ Max}_{cy}}\right) * \left( C_{\text{ optimal }}-C_i^t\right) \\&\quad \quad -\cos (\operatorname {rand}(0,1) * \pi ) \ * \left( \frac{t}{\text{ Max}_{cy}}\right) ^{\frac{2}{\text {Max}_{cy}}} * \left( C_i^t-C_{\text{ optimal }}\right) \\&\end{aligned} \end{aligned}$$

where $\operatorname {rand}(0,1) * \cos \left( 0.5 \pi * \frac{t}{\text{ Max}_{cy}}\right) * \left( C_{\text{ optimal }}-C_i^t\right)$ denotes the process of re-orientating to fly towards the master and $\cos (\operatorname {rand}(0,1) * \pi ) \ * \left( \frac{t}{\text{ Max}_{cy}}\right) ^{\frac{2}{\text {Max}_{cy}}} * \left( C_i^t-C_{\text{ optimal }}\right)$ indicates the procedure of moving away from the foreigners. The steps in the PO’s optimization methodology are represented as follows:

*Step 1:* Configure the parameters for the optimizer: Swarm size ($S_{in}$), individuals dimension (*D*), low bound (*lb*), high bound (*ub*), maximum number of cycles ($Max_{cy}$) and parameters ($\gamma$ and *Q*). *Step 2:* Initialize the parrots stochastically $(C_1, C_2, \ldots , C_{S_{in}})$. *Step 3:* Decide the parrots’ fitness (acquire host position and premium fitness). *Step 4:* While the stopping requirement was not met. **Step 5:** Update the swarm individuals using Eqs. (6), (9), (10), and (11), respectively, based on a random integer value called *St*, which takes a value from the discrete uniform distribution from 1 to 4 ($St = randi(1, 4)$). *Step 6:* If $St = 1$, then apply the foraging attitude using Eq. (6). *Step 7:* If $St = 2$, then apply the remaining attitude using Eq. (9). *Step 8:* If $St = 3$, then apply the communicating attitude using Eq. (10). *Step 9:* If $St = 4$, then apply the fear of strangers attitude using Eq. (11). *Step 10:* Apply boundary control on parrots and then update host position and premium fitness. **Step 11:** Return host position, premium fitness.

## The proposed multi-objective parrot optimizer (MOPO)

**Fig. 4:**
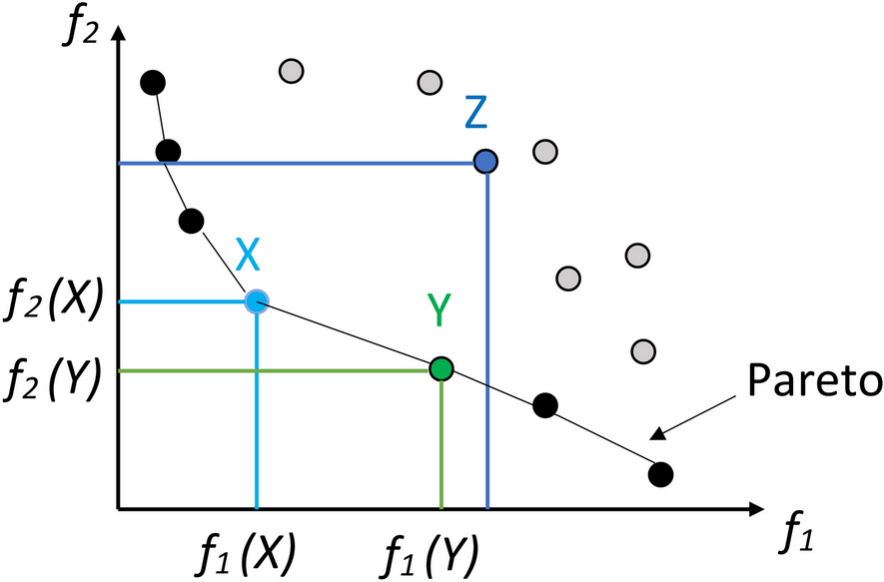
Illustration of Pareto optimal solutions (*X* and *Y*) dominating solution *Z* in a two-objective space.

To fully understand the MOPO approach, it’s important to explore the key mechanisms that it incorporates:

The process starts with an elitist non-dominated sorting method. This method identifies NDS within the search space and applies NDS and the non-dominated ranking (NDR) of these solutions^82^. Figure 4 illustrates how to use the NDR. While at least one solution from the first front dominates the solutions on the second front, none of the solutions on the first front do. According to Fig. 4, the NDR of a dominated solution in this situation denotes the number of solutions that defeat the solution *Z*^48^.

**Fig. 5:**
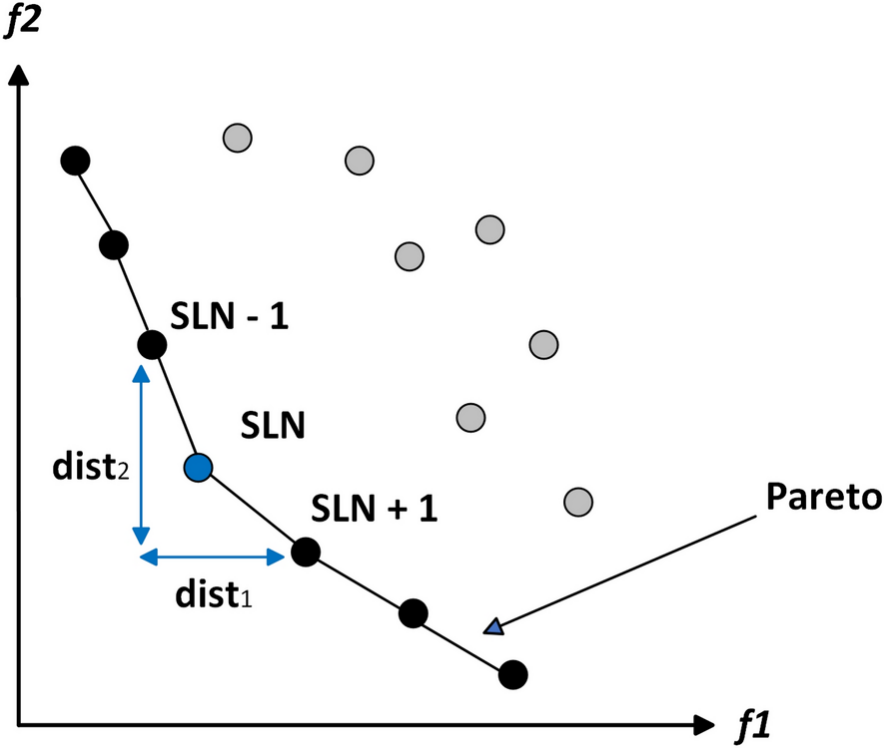
Crowding distance calculation process for solution SLN.

MOPO utilizes a CD operator to preserve solution variation. The CD operator gives an approximation of the density of the surrounding solutions. Figure 5 illustrates the CD for a solution *SLN*, defined as the size of the largest hexagonal area around *SLN* that does not contain any other solutions^48^. This strategy guarantees a balanced distribution of solutions within the search area. As shown in Eq. (12), the variety of solutions obtained by the CD operator is essential for avoiding premature convergence and guaranteeing the exploration of different regions in the search space.

12 $$\begin{aligned} { C D_{i}^{SLN}=\frac{f_{i}^{SLN+1}-f_{i}^{SLN-1}}{f_{i}^{high }-f_{i}^{low }} } \end{aligned}$$

where $f_{i}^{high}$ signifies the highest value of the *i*-th objective function, and $f_{i}^{low}$ denotes its lowest value. The CD for a solution *SLN* is calculated as the average distance between its neighboring solutions $(SLN-1, SLN+1)$.

**Fig. 6:**
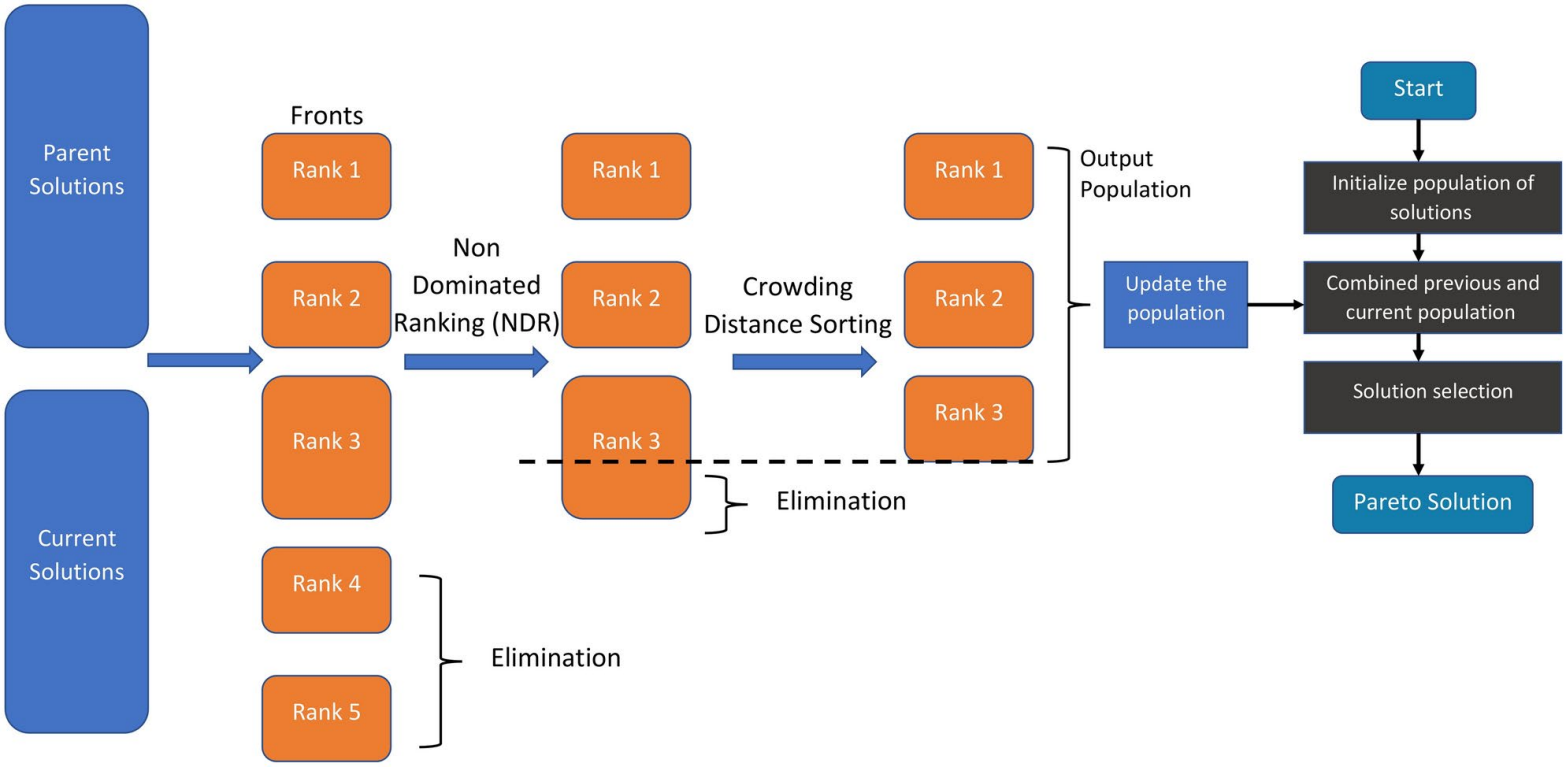
Clarification of non-dominated sorting approach.

The mechanism of the non-dominated sorting approach is depicted in Fig. 6. In the beginning, the parent and current population solutions use the NDR. After that, the solutions with the lowest rankings are removed, and the remaining solutions are arranged using the CD process. Only solutions with higher CD estimations are kept after those with the lowest CD values are eliminated. This process involves determining the target location dimension of the Pareto solution based on the CD value. The PO is then employed to update the positions of the solutions, with MOPO utilizing the same update process as the original PO. A detailed explanation of the PO’s optimization update process is provided in “The Parrot Optimizer (PO)” Section. Every solution in the population has its objective function assessed at each iteration, and the NDS are archived. This archive serves as the basis for the optimization process in the subsequent iteration.

The solutions stored in the archive are regularly updated to reflect the most recent changes, ensuring that outdated solutions are replaced. MOPO effectively refines solution distribution by utilizing the CD method, enabling it to achieve and archive Pareto optimal solutions. The optimization strategy for the developed MOPO is outlined in Algorithm 1. The MOO approach of the MOPO model can be summarized in the following steps:

*Step I*: Establish the control parameters required to make the optimizer execute. *Step II*: Randomly select the parent swarm $S_a$ within the decision space *S* using chaotic initialization. *Step III*: Within the objective space *F*, evaluate every objective function. *Step IV*: Generate a new swarm $S_b$ and combine it with $S_a$ to form the swarm $S_c$. *Step V*: Rank the solutions in $S_c$ based on NDS, incorporating both CD and NDR. *Step VI*: Populate the new candidate solution swarm with the top-performing $S_{in}$ solutions. *Step VII*: Repeat the above steps until the stopping criterion is satisfied.

The largest number of Pareto optimal solutions that may be stored in the archive, which is used in the MOO process, is constrained by the archive’s capacity. The rationale is that computation complexity increases with archive size. It is known that doing this reduces the actual approximation cost. Furthermore, the full archive issue can arise from reducing the archive size. To avoid these issues, the archive is periodically removed from neighborhoods with high population density, enabling freshly added solutions from areas with lower population density to be kept in the archive.

Ultimately, because the developed MOPO employs the same NDS and CD approaches as the NSGA-II algorithm, its computational complexity is $O(M * S_{in}^{2})$, where *M* is the number of target routines and $S_{in}$ indicates the swarm size. The following is an illustration of the computational time complexity for the biggest index of cycles:

13 $$\begin{aligned} \begin{array}{r} M=\left( \operatorname {D} * \operatorname {Max}_{\text{ cy }} * S_{in} + \operatorname {Cost}\left( f_{objective}\right) * \operatorname {Max}_{cy} * S_{\text{ in }} \right. \\ +(NDS + CD) * \left( \operatorname {Max}_{\text{ cy }}-\text{ t }\right) * \operatorname {D} + (NDS + CD) * \\ \left( \operatorname {Max}_{\text{ cy }}-\text{ t }\right) * \operatorname {Cost}\left( f_{objective}\right) \end{array} \end{aligned}$$

**Fig. 7:**
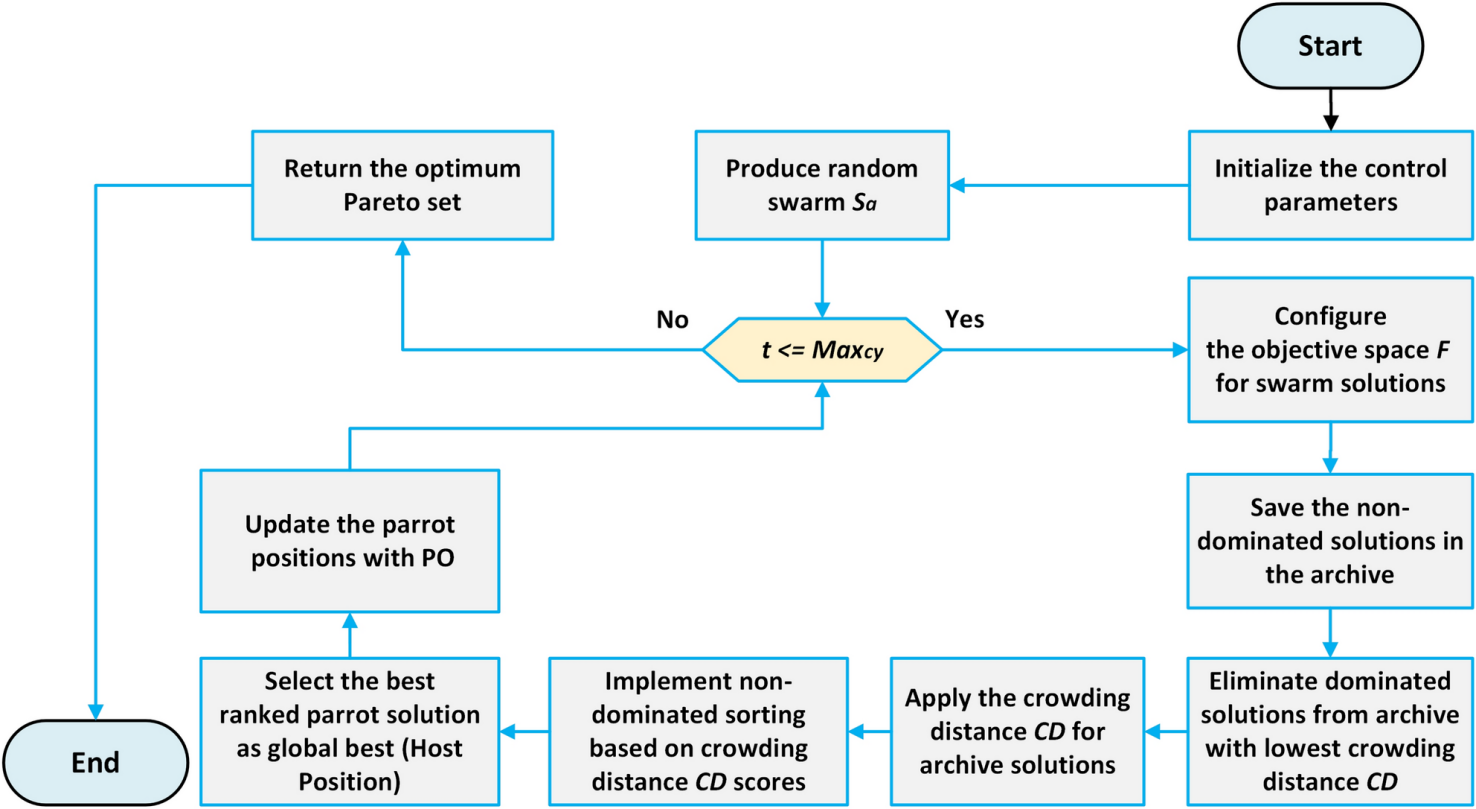
Flowchart of the developed MOPO

The flowchart for the developed MOPO is depicted in Fig. 7, where *t* represents the current cycle index and $Max_{cy}$ represents the largest index of cycles.

**Algorithm 1:**
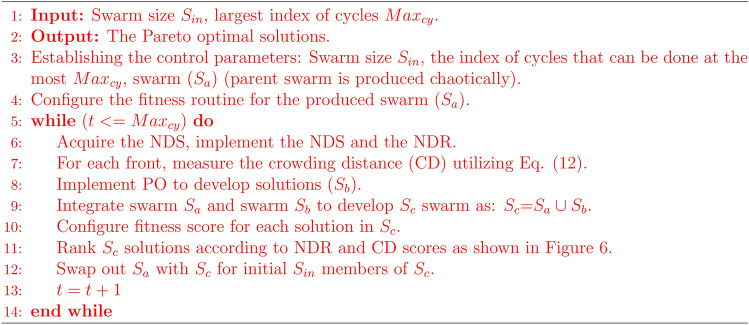
Pseudocode of the proposed MOPO algorithm.

## Excremental results analysis and discussion

This section describes how the CEC’2020 test routines were used to test and compare the suggested MOPO’s performance against other top algorithms. The evaluation and comparison of MOPSO^44^, NSGA-II^45^, MOGWO^46^, MOWOA^47^, MOSMA^48^, IMOMRFO^49^, and MOGTO^50^ reveal the performance of MOPO in the MOO domain.

### Experimental configuration

The experiments were performed in a standardized computing environment to ensure consistent and fair comparisons across all algorithms. The hardware setup includes an Intel Core i5-3317U CPU with a 1.70 GHz frequency, 4 GB RAM, and a 500 GB hard drive. The software environment consists of the Windows 8 operating system and MATLAB R2013a (8.1.0.604), which was used for implementing and running the optimization algorithms. These configurations were selected to provide a reliable and reproducible testing platform for evaluating the performance of the proposed MOPO algorithm against other state-of-the-art multi-objective optimization algorithms. All tests and simulations are executed on the same computer system to guarantee a fair comparison.

### Parameter configurations

To evaluate the robustness of the proposed MOPO technique on the CEC’2020 benchmark suite, this paper employs six standard performance metrics, comparing MOPO against other prominent methods^111^. Simulations are conducted using MATLAB R2013a as the integrated development environment (IDE). The results of MOPO are benchmarked against those from seven renowned multi-objective optimization algorithms. All algorithms are tested under standardized simulation settings to ensure a fair comparison. Table 1 details each method’s parameter settings and common configurations. Following best practices outlined in^112^, each method’s parameters were adjusted to their default configurations to minimize the risk of parameterization bias.

**Table 1:** Parameter configurations for MOPO and other competitive approaches.

Approach	Parameter settings
Shared configurations	
Swarm size	$S_{in}$ = 100
Maximum cycles	$Max_{cy}$ = 500
Independent runs	20
MOPSO	$\phi _{1} = \phi _{2}$ = 2.05, $\alpha$ = 0.1, $\beta$ = 4, *nGrid* = 10
NSGA-II	$P_c$ = 0.8, $P_m$ = 0.1
MOGWO	$nGrid = 10$, $\alpha$ = 0.1, $\beta$ = 10, $\gamma$ = 2
MOWOA	$archive_{size}$ = 100, *nGrid* = 10, $\alpha$ = 0.1, $\beta$ = 10, $\gamma$ = 2
MOSMA	$archive_{size}$ = 100, *z* = 0.03
IMOMRFO	$archive_{size}$ = 100, *Coef* = t/$Max_{cy}$, *S* = 2
MOGTO	$archive_{size}$ = 100, *p* = 0.03, *w* = 0.8, $\beta$ = 3
MOPO	$archive_{size}$ = 100, *Q* = rand(0, 1), $\gamma$ = 1.5

### Performance metrics

This paper uses six performance indicators to compare the proposed MOPO’s effectiveness versus rival algorithms: PSP, IGDX in decision space, HV, spacing, GD, and maximum spread. The PS generated, and the actual PS of the evaluation function are compared using the PSP metric^113^. This is how the PSP metric is computed in Eq. (14).

14 $$\begin{aligned} PSP=\frac{CR}{IGDX} \end{aligned}$$

where the cover rate is indicated by the *CR* measure, as indicated by Eq. (14). By calculating the arithmetic mean distance between the derived solutions of the method and the real solutions, IGDX defines the inverted generational distance in the solution domain^114^. Lower IGDX scores indicate more variability and integration. The IGDX metric is calculated as Eq. (15)

15 $$\begin{aligned} IGDX\left( U, U \right) =\frac{\sum _{v \in U } dist(v, U)}{\left| U^{*}\right| } \end{aligned}$$

where a set of acquired solutions is denoted by *U*, and a set of true/reference solutions is represented by $U^{*}$. The intersection proportion between the reference PS and the obtained PS is defined by the cover rate measure *CR*, which is computed as Eq. (16).

16 $$\begin{aligned} CR=\left( \prod _{l=1}^{n} \delta _{l}\right) ^{1 / 2n} \end{aligned}$$

where $\delta _{l}$ is calculated as follows Eq. (17):

17 $$\begin{aligned} { \delta _{l} =\left\{ \begin{array}{lc} 1 & V_{l}^{\max }=V_{l}^{\min } \\ 0 & v_{l}^{\min } \ge V_{l}^{\max } \Vert v_{l}^{\max } \le V_{l}^{\min } \\ \left( \frac{\min \left( v_{l}^{\max }, V_{l}^{\max }\right) -\max \left( v_{l}^{\min }, V_{l}^{\min }\right) }{V_{l}^{\max }-V_{l}^{\min }}\right) ^{2} & \text{ otherwise } \end{array}\right. } \end{aligned}$$

Here, *n* indicates the decision space’s dimensionality; $v_{l}^{\min }$ and $v_{l}^{\max }$ represent the minimum and max of the acquired PS for the *l*th parameter. In contrast, $V_{l}^{\min }$ and $V_{l}^{\max }$ indicate the true PS for the *l*th variable^113^.

The HV metric estimates the extent of the objective space dominated by approximated solutions *SLN* and restricted by a reference point $Ref=\left( Ref_{1}, \ldots , Ref_{u}\right) ^{T}$, which is defeated by all attributes on the PF^61^. It is calculated by Eq. (18)

18 $$\begin{aligned} H V(SLN)=\operatorname {L}\left( \cup _{a \in SLN}\left[ f_{1}(a), Ref_{1}\right] \times \cdots \times \left[ f_{u}(a), Ref_{u}\right] \right) \end{aligned}$$

where the Lebesgue metric of a set *Z* is denoted by $\operatorname {L}(Z)$. For bi-objective test routines, *Ref* is typically set to $(1.2,1.2)^{T}$, and for three-objective test routines, to $(1.2,1.2,1.2)^{T}$.

The Spacing is calculated by Eq. (19).

19 $$\begin{aligned} \text{ Spacing }=\sqrt{\frac{1}{n-1} \sum _{i=1}^n\left( \bar{c}-d_i\right) ^2} \end{aligned}$$

The Generational distance (GD) is calculated in Eq. (20):

20 $$\begin{aligned} \text{GD}=\frac{\sqrt{\sum _{i=1}^{m} d_i^2}}{n} \end{aligned}$$

The Maximum spread is calculated by Eq. (21).

21 $$\begin{aligned} \text{ Maximum } \text{ Spread }=\sqrt{\sum _{i=1}^o \max \left( d\left( a_i, b_i\right) \right) } \end{aligned}$$

where *n* denotes the number of Pareto optimal solutions, $d_i$ represents the Euclidean distance between the *i*th real Pareto optimal solution and the nearest solution found, *m* denotes the number of actual Pareto optimal solutions, and $\bar{c}$ represents the average of all $d_i$. $d_i=\min _j\left( \left| f_1^i(\vec {x})-f_1^j(\vec {x})\right| +\left| f_2^i(\vec {x})-f_2^j(\vec {x})\right| \right)$ for $i, j=1,2, \ldots , n$, where $f_1^i(\vec {x})$ represents the objective value of the *i*th real Pareto optimal solution, and $f_1^j(\vec {x})$ represents the objective value of the closest *j*th Pareto optimal solution obtained in the reference set.

To sum up, greater algorithmic efficacy is indicated by high PSP, low IGDX, high HV, low spacing, low GD, and low spread.

###  Series 1: analysis of MO CEC’2020 functions

**Fig. 8:**
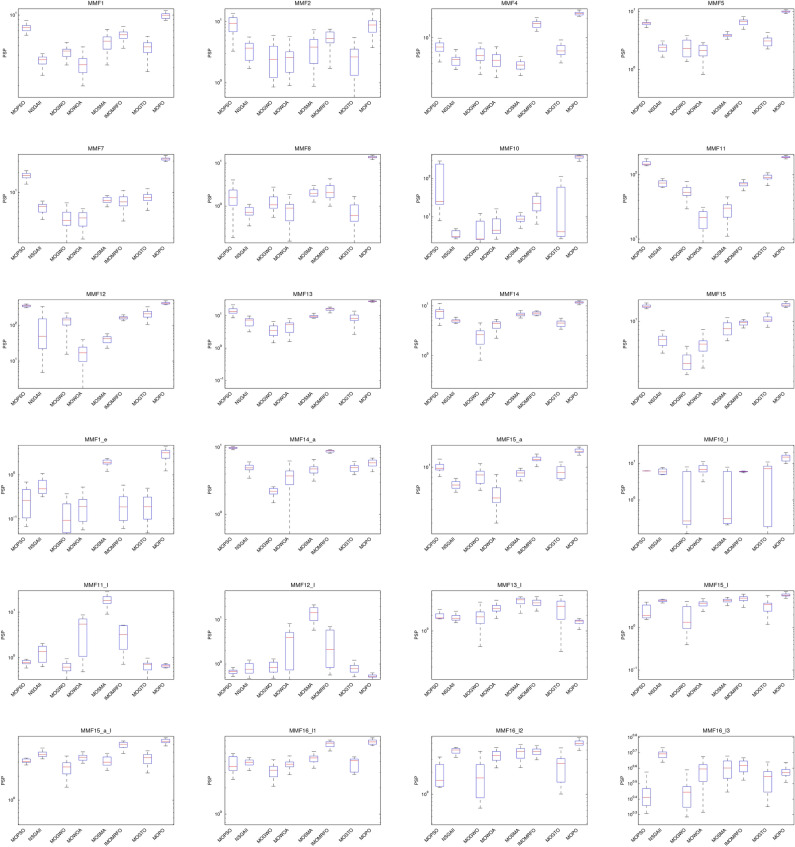
Boxplot for MOPO and other MO algorithms on 24 test function.

The CEC’2020 benchmarks contain 24 assessment cases with varying multi-objective decision regions (featuring nonlinear, linear, convex, and concave PF topologies), which are used in this paper to evaluate the effectiveness of the proposed MOPO technique. The competing algorithms were tested over 20 independent runs, each consisting of 500 iterations, 50,000 function evaluations, and 100 individuals per test case. The results are presented as average (Mean) and standard deviation (SD) statistical indicators in Tables 2, 3 and 4. Additionally, Fig. 8 illustrates the boxplot of the PSP measure for the 24 test cases from the CEC’2020 benchmark. For this reason, the highest PSP score denotes better algorithm performance because, as Eq. (14) illustrates, the PSP metric is a maximization metric.

Boxplot graphs are considered the most significant method of illustrating the agreement between the presented data, even though they describe the data distribution. Figure 8 makes it evident that except MMF14_a, MMF11_I, MMF12_I, MMF13_I, and MMF16_l3, the boxplots of the developed MOPO technique are tight and have the highest scores for the majority of test routines. MOSMA does better in MMF11_I and MMF12_I than MOPO. MOPSO, IMOMRFO, and NSGA-II outperform MOPO in MMF14_a, MMF13_I, and MMF16_l3, respectively. Thus, we can conclude that the MOPO only performs mediocrely in MMF14_a, MMF11_I, MMF12_I, MMF13_I, and MMF16_l3. Generally, in most evaluation routines, the MOPSO, NSGA-II, and MOGWO algorithms yield subpar results with the lowest PSP scores; similarly, the MOWOA in certain test routines, notably MMF7, MMF11, and MMF12, exhibit poor performance.

The statistical findings of the PSP metric on 20 runs for each algorithm are presented in Table 2 as Mean and SD. Mostly, the MOPO produces the greatest results regarding evaluation concerns. But with MMF14_a, the MOPSO performs better than the MOPO. Furthermore, using MMF11_I and MMF12_I, the MOSMA performs better than the MOPO. Likewise, with MMF13_I and MMF16_l3, respectively, the IMOMRFO and the NSGA-II perform better than the developed MOPO. Similarly, Table 4 presents statistical findings for the IGDX metric for 20 runs of each algorithm, broken down into Mean and SD. In most test routines, the MOPO typically yields the best results. But with MMF14_a, the MOPSO performs better than the MOPO. Furthermore, using MMF11_I and MMF12_I, the MOSMA performs better than the MOPO. Likewise, with MMF13_I and MMF16_l3, the IMOMRFO and the NSGA-II perform better than the developed MOPO. Similarly, Table 4 shows the statistical findings of the HV metric in terms of Mean and SD over 20 runs for each algorithm. In general, the developed MOPO outperforms most test methods. However, the MOPSO beats the MOPO regarding MMF14_a, similar to the PSP and IGDX findings. Furthermore, the MOSMA outperforms the MOPO in MMF11_I and MMF12_I. Furthermore, the IMOMRFO and the NSGA-II outperform the developed MOPO using MMF13_I and MMF16_l3, respectively. After this point, focusing on the tabulated PSP, IGDX, and HV statistical findings, it can be stated that when using the PSP, IGDX, and HV measurements, the produced MOPO produces superior results in most evaluation cases. Still, it may rank second or third in its poorest performance.

**Fig. 9:**
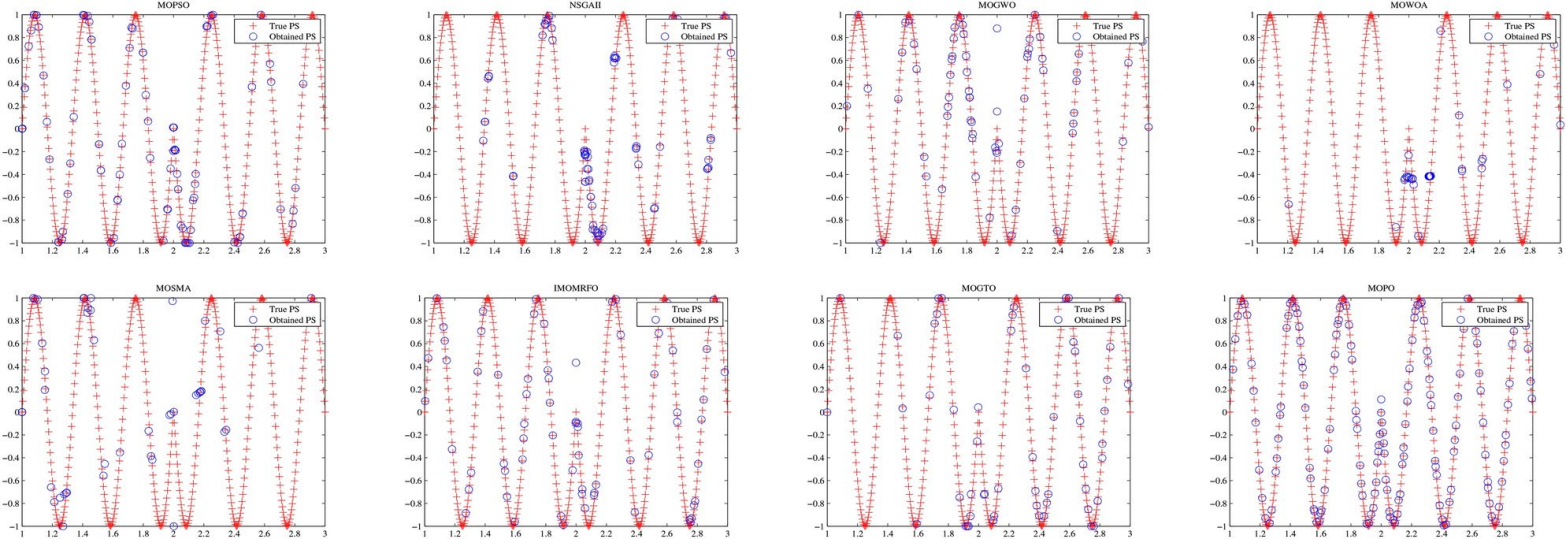
The PS for MMF1 test function.

Convergence curves offer a graphical representation essential for assessing the Pareto solutions (PSs) generated by different algorithms. Figure 9 through 11 illustrate the convergence behavior of the evaluated methods across various test routines. Specifically, routines MMF1, MMF14, and MMF15_a have been selected for detailed visualization to validate the PSs produced by the algorithms in question.

**Fig. 10:**
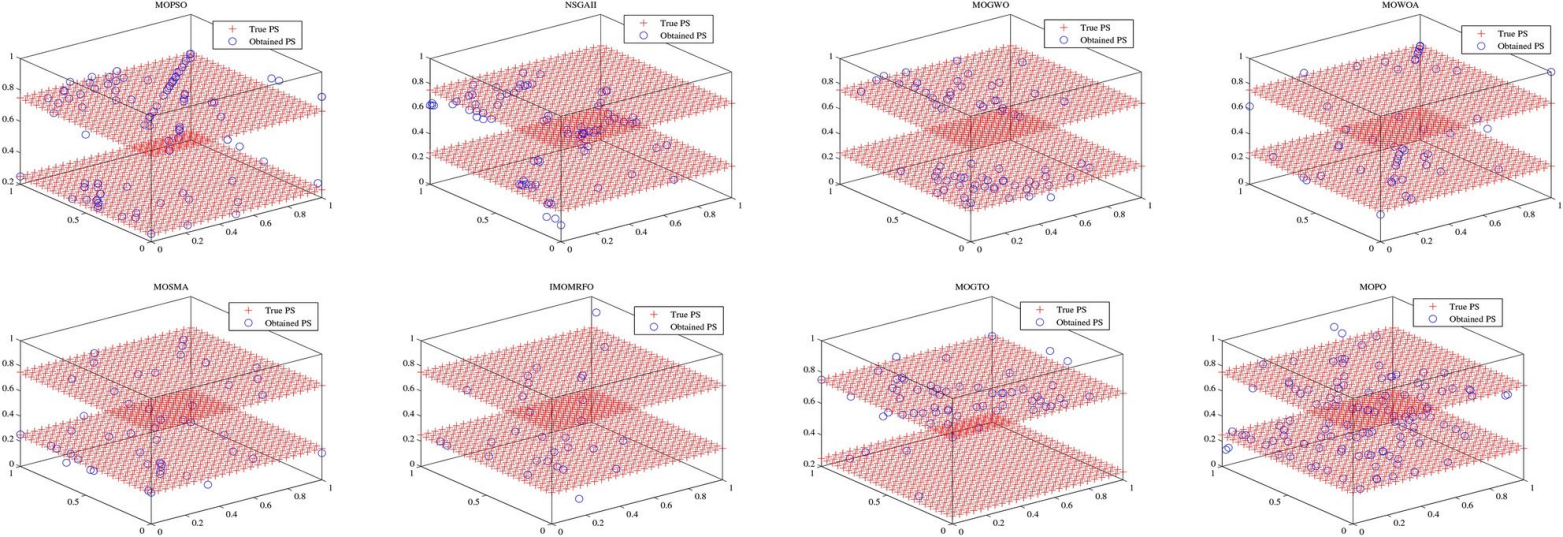
The PS for MMF14 test function.

Figure 9 reveals that the MOPO algorithm consistently produces superior PSs compared to the other methods under review. In Fig. 10, it is evident that MOPO excels in locating more PSs within the decision space compared to the seven competing approaches. Moreover, compared to NSGA-II, MOWOA, and MOSMA, the PSs generated by MOPO are more evenly distributed throughout the global PSs in the test case space.

**Fig. 11:**
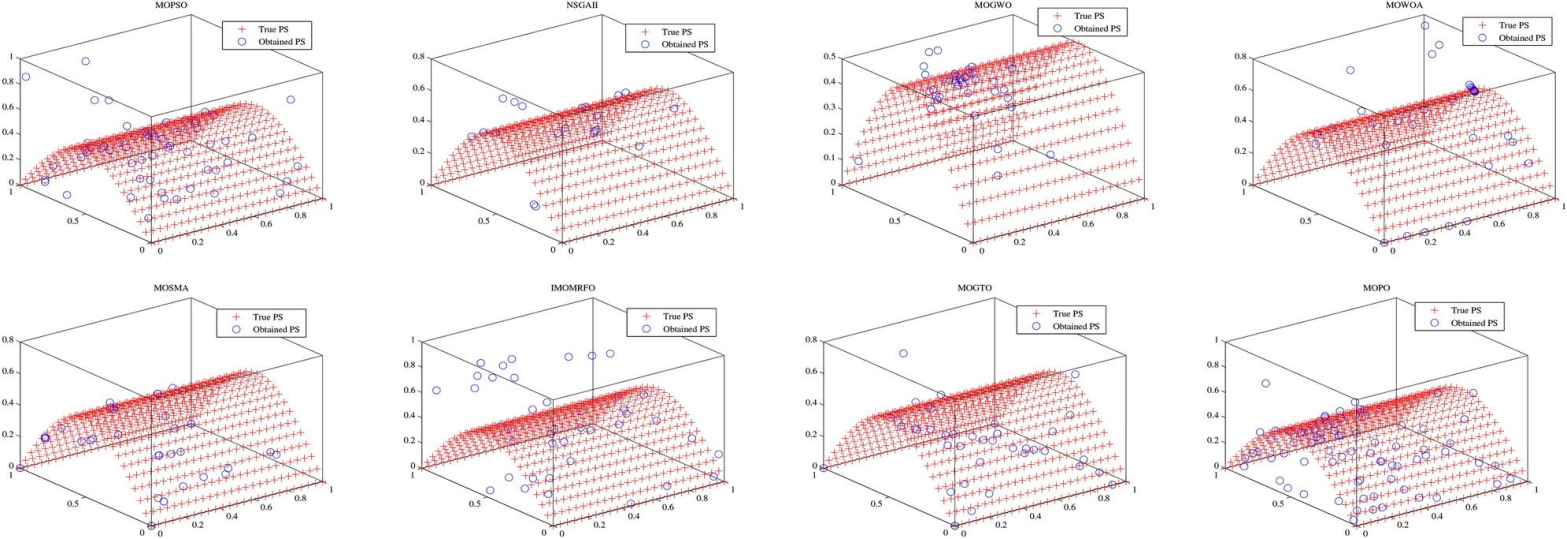
Obtained PS for MMF15_a test function.

Figure 11 presents the PSs for the MMF15_a routine. Here, MOPO, MOPSO, MOSMA, and MOGTO demonstrate its capability to generate additional PSs in the decision space. Notably, MOPO’s PSs are more uniformly spread than its competitors.

**Fig. 12:**
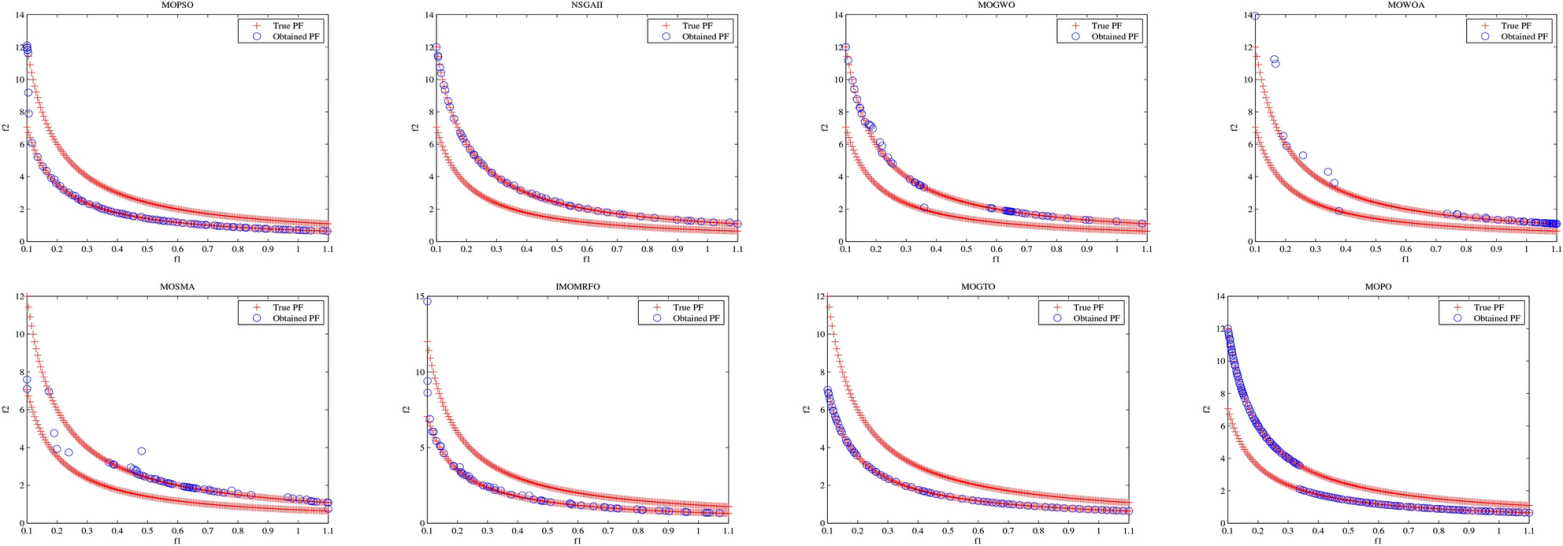
The PF for MMF10-I test function.

In contrast, Figures 12 through 14 display the convergence curves of the Pareto fronts (PFs) for the MMF10_I, MMF12, and MMF15_a test routines. Figure 12 shows that the PF sets obtained by MOPSO, IMOMRFO, and MOGTO are significantly larger than those of other methods. However, MOPO achieves a notably superior PF compared to its peers.

**Fig. 13:**
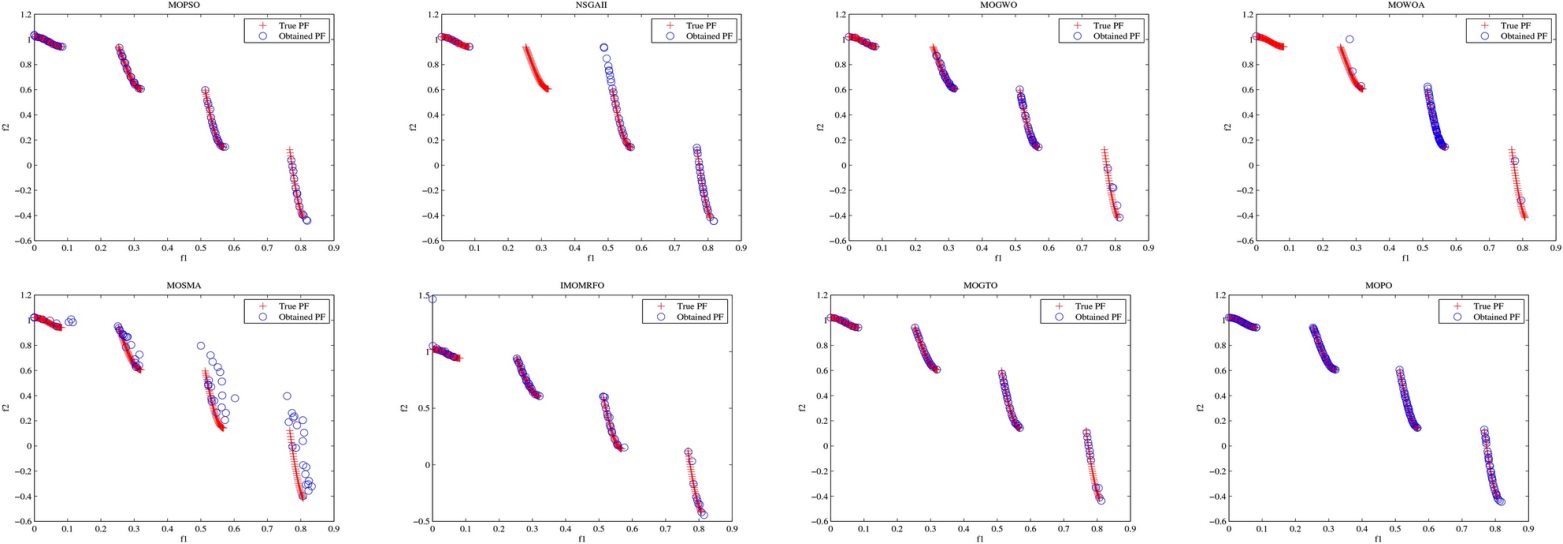
The PF for MMF12 test function.

**Fig. 14:**
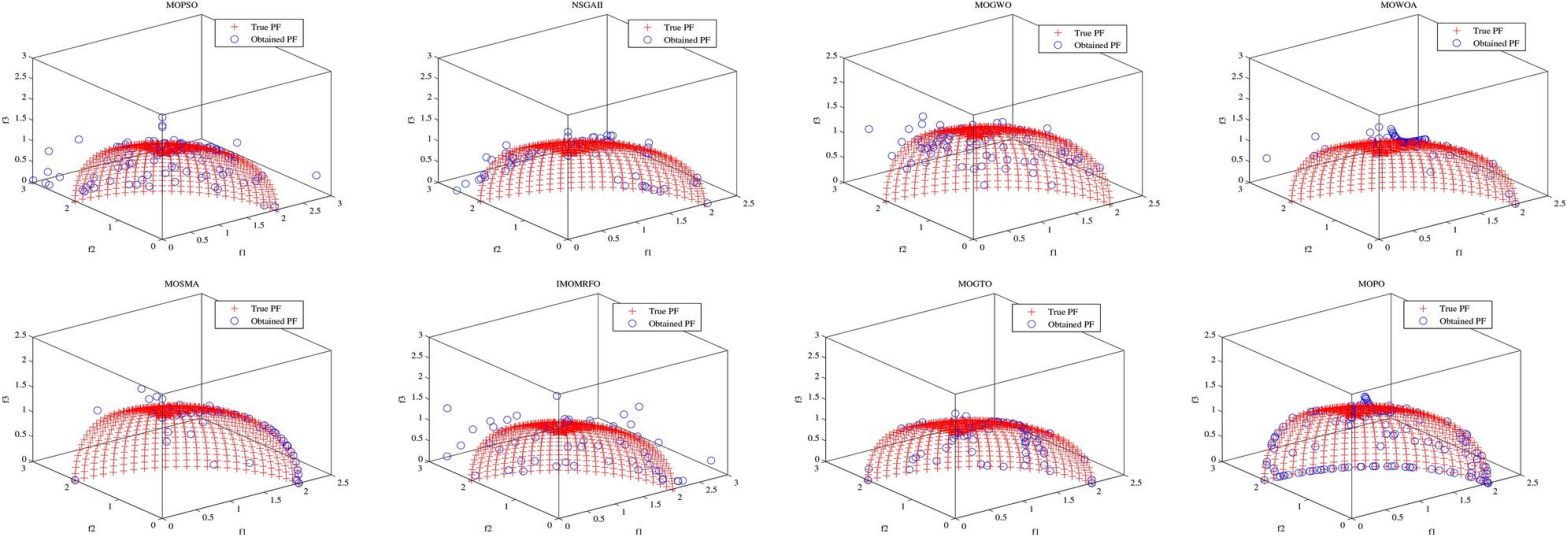
The PF for MMF15_a test function.

Figure 13 illustrates the PF for the MMF12 function, where MOPO stands out by generating a more uniformly distributed PF across the objective space compared to NSGA-II, MOGWO, MOWOA, and MOSMA. Similarly, Fig. 14 demonstrates that MOSMA, MOGTO, and MOPO are particularly effective in identifying larger and more evenly distributed PFs in the objective space relative to their competitors.

The Wilcoxon rank-sum test is also used to conduct statistical analysis and confirm the significance of the observed results. Because metaheuristic algorithms are inherently unpredictable and stochastic, it is important to ensure that the performance outcomes do not result from chance^115^. Table 5 presents the results of the Wilcoxon test for each pair of algorithms in the PSs comparison. The MOPO’s performance is confirmed to be significant, with all *p*-values being $\le$ 0.05, which is below the 5% significance level for the PSP, IGDX, and HV metrics.

**Table 2:** Comparison of PSP Values for MOPO and Other Multi-Objective Algorithms.

PSP		MOPSO	NSGA-II	MOGWO	MOWOA	MOSMA	IMOMRFO	MOGTO	MOPO
MMF1	Mean	7.47	3.50	4.17	3.29	5.35	6.37	4.72	**9.81**
	SD	0.64	0.47	0.73	0.74	1.01	0.76	0.90	**0.83**
MMF2	Mean	8.72	3.54	2.70	2.57	3.81	5.41	2.60	**8.93**
	SD	3.21	1.21	1.62	1.31	2.01	2.12	1.38	**2.89**
MMF4	Mean	7.38	4.53	5.52	4.52	3.82	16.35	6.50	**23.70**
	SD	1.84	0.88	1.47	1.23	0.74	2.15	1.49	**1.61**
MMF5	Mean	6.17	2.36	2.43	2.08	3.89	6.56	3.11	**9.89**
	SD	0.51	0.39	0.82	0.56	0.34	0.85	0.63	**0.46**
MMF7	Mean	18.11	5.82	4.06	3.96	7.56	7.39	8.27	**32.36**
	SD	2.14	0.96	1.54	1.08	0.92	1.74	1.56	**1.74**
MMF8	Mean	1.87	0.74	1.28	0.84	2.04	2.25	0.73	**13.77**
	SD	1.22	0.22	0.62	0.47	0.59	0.96	0.39	**0.99**
MMF10	Mean	98.02	5.13	5.64	6.65	9.08	23.26	30.61	**360.34**
	SD	110.25	7.02	5.13	3.94	2.80	11.36	42.81	**47.68**
MMF11	Mean	153.06	64.89	53.97	20.88	29.07	71.31	90.10	**187.01**
	SD	17.27	24.81	12.72	7.29	9.04	6.91	10.15	**6.24**
MMF12	Mean	347.65	93.47	130.69	18.09	41.95	162.65	210.27	**420.29**
	SD	64.69	95.92	58.51	10.95	13.49	16.12	61.06	**33.09**
MMF13	Mean	14.13	6.77	3.85	4.87	9.60	15.83	8.73	**27.85**
	SD	3.61	1.93	1.88	1.97	0.90	1.45	3.07	**1.14**
MMF14	Mean	7.19	4.96	2.59	4.16	6.74	7.11	4.45	**11.74**
	SD	2.04	0.58	1.07	0.79	0.65	0.53	0.69	**0.55**
MMF15	Mean	14.99	6.01	3.44	5.31	8.30	9.58	10.60	**15.53**
	SD	0.97	0.99	0.85	1.31	1.63	0.61	1.00	**0.70**
MMF1_e	Mean	0.34	0.57	0.14	0.21	1.89	0.30	0.21	**3.11**
	SD	0.27	0.22	0.11	0.13	0.34	0.36	0.13	**0.88**
MMF14_a	Mean	**9.67**	4.96	2.26	3.53	4.79	8.74	4.88	5.78
	SD	**0.41**	0.63	0.45	1.71	1.00	0.37	0.63	0.77
MMF15_a	Mean	9.86	6.75	8.19	5.72	8.74	11.87	9.10	**14.21**
	SD	1.17	0.77	1.37	1.81	0.67	0.82	1.24	**0.69**
MMF10_I	Mean	5.59	5.54	2.72	7.17	2.28	5.69	5.55	**15.13**
	SD	1.84	2.06	3.05	2.25	3.19	0.35	3.80	**5.09**
MMF11_I	Mean	1.45	1.86	0.87	4.71	**18.30**	3.25	0.67	0.66
	SD	1.63	1.77	1.12	2.89	**4.57**	1.77	0.15	0.05
MMF12_I	Mean	0.99	1.01	1.25	3.48	**16.38**	3.07	0.79	0.52
	SD	1.41	0.97	1.29	2.63	**9.90**	2.48	0.26	0.07
MMF13_I	Mean	2.11	1.97	1.98	2.81	3.79	**3.87**	2.86	1.44
	SD	0.66	0.57	0.89	0.54	0.72	**0.72**	1.24	0.33
MMF15_I	Mean	2.40	4.29	1.97	3.66	4.29	4.95	3.19	**5.92**
	SD	0.94	0.45	1.20	0.65	0.65	0.85	1.08	**0.55**
MMF15_a_I	Mean	3.26	3.77	2.52	3.38	3.05	4.87	3.29	**5.46**
	SD	0.49	0.31	0.69	0.59	0.48	0.55	0.58	**0.39**
MMF16_l1	Mean	3.68	3.87	3.14	3.73	4.33	6.23	3.73	**6.71**
	SD	0.76	0.32	0.57	0.43	0.46	0.71	0.84	**0.40**
MMF16_l2	Mean	1.86	3.93	1.79	3.39	3.68	3.80	2.40	**5.00**
	SD	0.65	0.36	1.01	0.59	0.71	0.76	1.01	**0.52**
MMF16_l3	Mean	2.66	**4.87**	2.71	3.80	3.90	3.75	3.37	4.09
	SD	0.48	**0.29**	0.54	0.90	0.58	0.40	0.65	0.28
Friedman mean rank		5.16	3.85	2.29	3.04	4.64	6.04	3.95	7.00
Rank		3	6	8	7	4	2	5	1

Significant values are in (bold).

**Table 3:** IGDX Metric Values for MOPO Compared to Other Multi-Objective Algorithms.

IGDX		MOPSO	NSGA-II	MOGWO	MOWOA	MOSMA	IMOMRFO	MOGTO	MOPO
MMF1	Mean	0.13	0.28	0.23	0.29	0.19	0.15	0.21	**0.10**
	SD	0.01	0.05	0.04	0.06	0.04	0.02	0.04	**0.01**
MMF2	Mean	0.13	0.28	0.33	0.34	0.27	0.17	0.38	**0.12**
	SD	0.05	0.10	0.12	0.10	0.11	0.06	0.14	**0.05**
MMF4	Mean	0.14	0.23	0.18	0.22	0.26	0.06	0.16	**0.04**
	SD	0.03	0.04	0.05	0.06	0.05	0.01	0.04	**0.00**
MMF5	Mean	0.16	0.43	0.39	0.46	0.25	0.15	0.32	**0.10**
	SD	0.01	0.08	0.09	0.11	0.02	0.02	0.06	**0.01**
MMF7	Mean	0.05	0.18	0.16	0.19	0.13	0.10	0.11	**0.03**
	SD	0.01	0.03	0.03	0.05	0.01	0.01	0.01	**0.00**
MMF8	Mean	0.71	1.48	0.75	1.22	0.49	0.44	1.27	**0.07**
	SD	0.54	0.54	0.28	0.67	0.13	0.16	0.52	**0.01**
MMF10	Mean	0.04	0.29	0.28	0.19	0.12	0.05	0.19	**0.00**
	SD	0.03	0.09	0.14	0.09	0.04	0.04	0.15	**0.00**
MMF11	Mean	0.01	0.03	0.02	0.05	0.04	0.01	0.01	**0.01**
	SD	0.00	0.04	0.01	0.02	0.02	0.00	0.00	**0.00**
MMF12	Mean	0.00	0.04	0.01	0.08	0.03	0.01	0.01	**0.00**
	SD	0.00	0.05	0.02	0.06	0.01	0.00	0.00	**0.00**
MMF13	Mean	0.08	0.16	0.13	0.21	0.10	0.06	0.11	**0.04**
	SD	0.02	0.05	0.02	0.08	0.01	0.00	0.03	**0.00**
MMF14	Mean	0.15	0.20	0.32	0.25	0.15	0.14	0.23	**0.09**
	SD	0.05	0.03	0.07	0.05	0.01	0.01	0.04	**0.00**
MMF15	Mean	0.07	0.17	0.20	0.20	0.12	0.10	0.10	**0.06**
	SD	0.00	0.03	0.03	0.04	0.02	0.01	0.01	**0.00**
MMF1_e	Mean	2.20	1.88	3.00	2.69	0.54	2.44	2.53	**0.33**
	SD	0.90	0.67	0.63	0.70	0.12	0.94	0.75	**0.10**
MMF14_a	Mean	**0.10**	0.20	0.30	0.31	0.22	0.11	0.21	0.18
	SD	**0.00**	0.03	0.02	0.14	0.04	0.00	0.03	0.03
MMF15_a	Mean	0.10	0.15	0.12	0.19	0.12	0.08	0.11	**0.07**
	SD	0.01	0.02	0.02	0.05	0.01	0.01	0.01	**0.00**
MMF10_I	Mean	0.19	0.17	0.19	0.14	0.15	0.17	0.15	**0.13**
	SD	0.04	0.03	0.03	0.03	0.02	0.00	0.04	**0.01**
MMF11_I	Mean	0.24	0.19	0.25	0.19	**0.06**	0.22	0.25	0.25
	SD	0.02	0.07	0.02	0.06	**0.02**	0.02	0.00	0.00
MMF12_I	Mean	0.24	0.22	0.24	0.23	**0.08**	0.21	0.25	0.20
	SD	0.02	0.06	0.02	0.06	**0.04**	0.04	0.00	0.00
MMF13_I	Mean	0.26	0.28	0.30	0.31	0.23	**0.22**	0.27	0.25
	SD	0.02	0.03	0.04	0.05	0.02	**0.03**	0.06	0.03
MMF15_I	Mean	0.27	0.24	0.33	0.27	0.23	0.20	0.28	**0.17**
	SD	0.01	0.03	0.06	0.04	0.03	0.03	0.04	**0.02**
MMF15_a_I	Mean	0.26	0.26	0.30	0.29	0.30	0.20	0.29	**0.18**
	SD	0.02	0.02	0.03	0.04	0.03	0.02	0.03	**0.01**
MMF16_l1	Mean	0.23	0.26	0.27	0.27	0.23	0.16	0.25	**0.15**
	SD	0.02	0.02	0.02	0.03	0.03	0.02	0.03	**0.01**
MMF16_l2	Mean	0.34	0.26	0.37	0.29	0.26	0.25	0.33	**0.20**
	SD	0.02	0.03	0.06	0.04	0.04	0.03	0.05	**0.02**
MMF16_l3	Mean	0.29	**0.21**	0.30	3.80	0.25	0.27	0.27	0.24
	SD	0.03	**0.01**	0.02	0.90	0.03	0.02	0.03	0.02
Friedman mean rank		3.62	5.41	6.58	6.62	4.12	2.79	5.25	1.58
Rank		3	6	7	8	4	2	5	1

Significant values are in (bold).

**Table 4:** HV Metric Values for MOPO and Competing Multi-Objective Algorithms.

HV		MOPSO	NSGA-II	MOGWO	MOWOA	MOSMA	IMOMRFO	MOGTO	MOPO
MMF1	Mean	0.85	0.83	0.83	0.81	0.46	0.85	0.84	**0.87**
	SD	0.01	0.01	0.01	0.02	0.22	0.00	0.01	**0.00**
MMF2	Mean	0.46	0.64	0.70	0.57	0.65	0.68	0.61	**0.83**
	SD	0.08	0.07	0.07	0.09	0.08	0.05	0.10	**0.01**
MMF4	Mean	0.53	0.51	0.51	0.49	0.49	0.53	0.52	**0.54**
	SD	0.00	0.02	0.01	0.02	0.01	0.00	0.00	**0.00**
MMF5	Mean	0.87	0.83	0.84	0.82	0.34	0.87	0.84	**0.87**
	SD	0.00	0.01	0.01	0.02	0.22	0.00	0.01	**0.00**
MMF7	Mean	0.87	0.85	0.85	0.84	0.74	0.85	0.86	**0.87**
	SD	0.00	0.01	0.00	0.01	0.05	0.00	0.00	**0.00**
MMF8	Mean	0.42	0.41	0.39	0.37	0.38	0.39	0.41	**0.42**
	SD	0.00	0.00	0.04	0.02	0.04	0.02	0.00	**0.00**
MMF10	Mean	12.64	11.65	11.73	11.29	11.51	12.28	12.04	**12.83**
	SD	0.19	0.27	0.44	0.38	0.20	0.20	0.53	**0.02**
MMF11	Mean	14.50	14.37	14.36	13.76	13.90	14.37	14.45	**14.50**
	SD	0.00	0.15	0.04	0.38	0.21	0.03	0.01	**0.00**
MMF12	Mean	1.57	1.41	1.48	1.04	0.81	1.55	1.55	**1.57**
	SD	0.00	0.17	0.15	0.30	0.22	0.01	0.03	**0.00**
MMF13	Mean	18.40	18.08	18.20	17.32	17.82	18.24	18.32	**18.41**
	SD	0.01	0.30	0.04	0.26	0.32	0.02	0.07	**0.00**
MMF14	Mean	2.64	2.67	2.24	2.67	2.90	2.62	2.82	**3.09**
	SD	0.26	0.35	0.39	0.78	0.32	0.34	0.18	**0.14**
MMF15	Mean	4.01	3.61	3.22	3.93	4.25	3.86	4.23	**4.27**
	SD	0.18	0.31	0.39	0.78	0.51	0.36	0.17	**0.12**
MMF1_e	Mean	0.86	0.76	0.83	0.57	0.53	0.86	0.82	**0.86**
	SD	0.01	0.07	0.04	0.38	0.09	0.01	0.04	**0.01**
MMF14_a	Mean	**3.07**	2.93	2.15	2.85	2.90	2.71	2.96	3.00
	SD	**0.17**	0.09	0.31	0.82	0.26	0.32	0.25	0.25
MMF15_a	Mean	4.06	4.02	3.98	4.73	4.36	4.08	4.58	**4.65**
	SD	0.15	0.21	0.24	0.86	0.36	0.26	0.40	**0.18**
MMF10_I	Mean	12.66	11.98	12.55	11.74	11.73	12.73	12.19	**12.85**
	SD	0.16	0.40	0.29	0.29	0.13	0.05	0.47	**0.01**
MMF11_I	Mean	14.48	14.32	14.32	13.77	**14.56**	14.46	14.44	14.49
	SD	0.01	0.13	0.05	0.28	**0.19**	0.01	0.02	0.00
MMF12_I	Mean	1.55	1.33	1.44	0.93	**1.57**	1.53	1.52	1.56
	SD	0.00	0.27	0.16	0.23	**0.30**	0.00	0.07	0.00
MMF13_I	Mean	18.39	17.28	17.77	16.84	17.19	**18.95**	17.78	18.40
	SD	0.01	0.28	0.27	0.31	0.27	**0.17**	0.30	0.01
MMF15_I	Mean	3.92	3.45	3.37	4.04	4.21	4.05	4.24	**4.33**
	SD	0.18	0.50	0.60	0.71	0.64	0.25	0.53	**0.12**
MMF15_a_I	Mean	4.00	3.70	3.34	3.83	4.46	3.98	4.35	**4.59**
	SD	0.17	0.53	0.57	0.76	0.46	0.43	0.46	**0.27**
MMF16_l1	Mean	4.04	3.88	3.81	3.71	4.19	4.06	4.12	**4.32**
	SD	0.23	0.64	0.66	0.86	0.64	0.24	0.42	**0.19**
MMF16_l2	Mean	3.97	3.59	3.56	4.12	3.99	3.96	4.26	**4.28**
	SD	0.20	0.48	0.60	0.87	0.48	0.30	0.38	**0.13**
MMF16_l3	Mean	3.80	**4.56**	3.64	3.78	4.02	3.72	4.23	4.42
	SD	0.33	**0.16**	0.53	0.51	0.56	0.44	0.36	0.41
Friedman mean rank		5.62	3.37	2.93	2.45	3.81	4.85	5.35	7.58
Rank		2	6	7	8	5	4	3	1

Significant values are in (bold).

**Table 5:** Wilcoxon Test Results for MOPO and Other MO algorithms on PSP, IGDX, and HV Metrics.

Test Metric	MOPO versus MOPSO	MOPO versus NSGA-II	MOPO versus MOGWO	MOPO versus MOWOA	MOPO versus MOSMA	MOPO versus IMOMRFO	MOPO versus MOGTO
PSP	1.82E−04	4.15E−05	2.00E−05	1.05E−04	9.45E−04	5.22E−04	2.56E−05
IGDX	2.04E−04	4.38E−05	1.39E−05	1.66E−05	9.84E−04	1.20E−03	2.07E−05
HV	3.26E−04	2.89E−05	9.37E−06	2.00E−05	2.00E−05	2.33E−04	9.32E−06

###  Series 2: analysis of standard MO problems

**Fig. 15:**
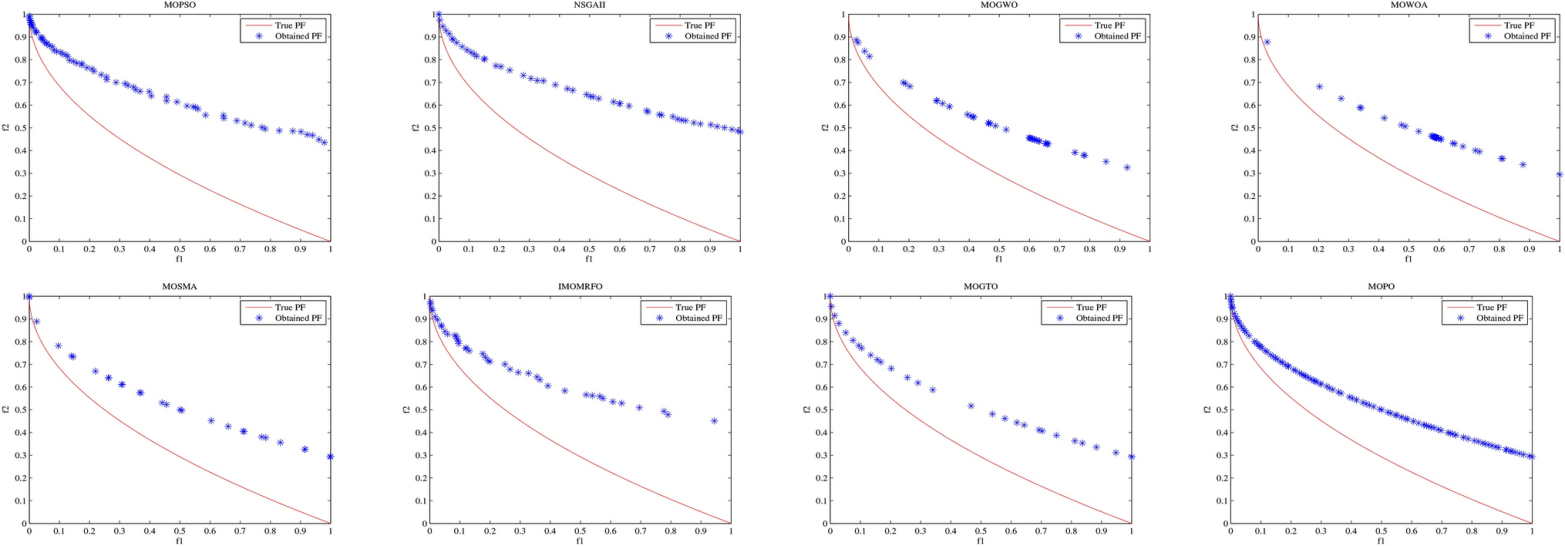
Pareto front acquired for ZDT1 function.

**Fig. 16:**
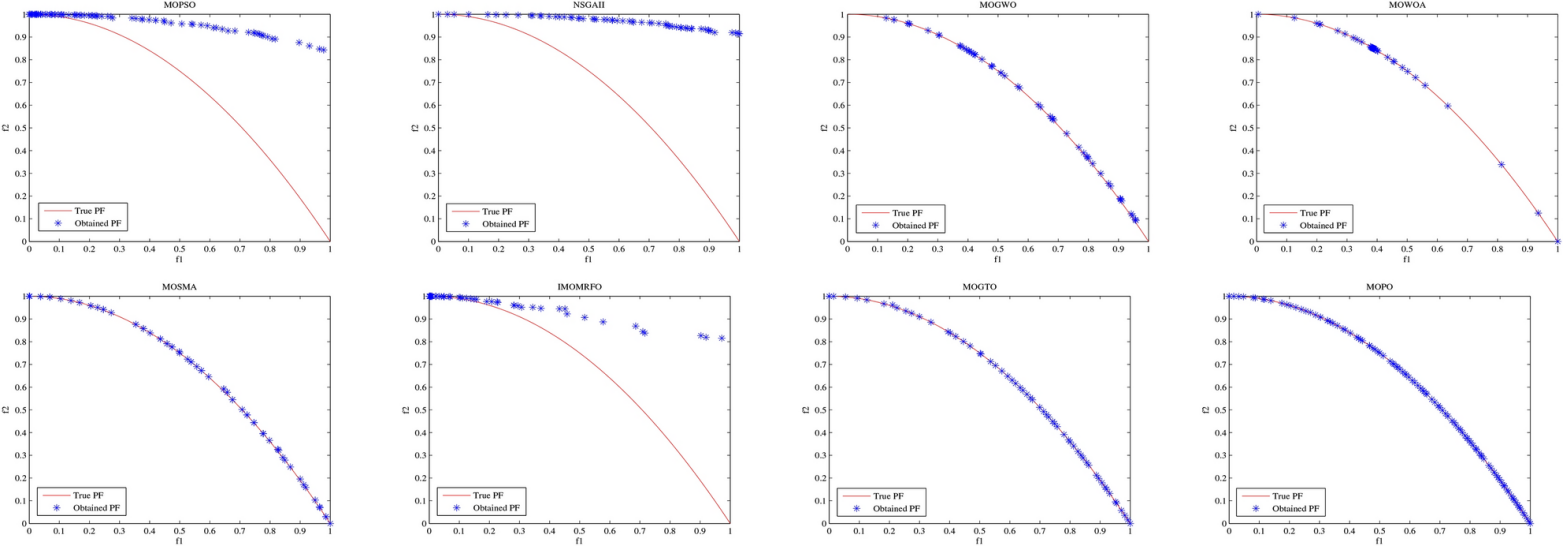
Pareto front obtained for ZDT2 test function.

**Fig. 17:**
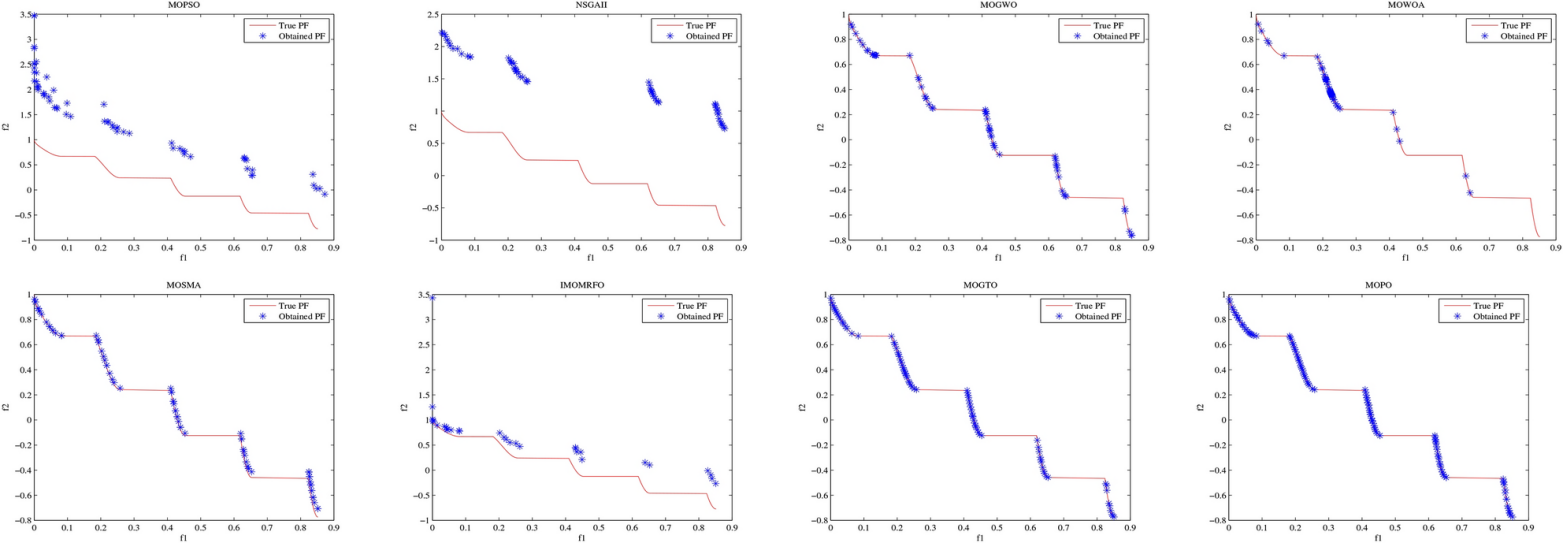
Pareto front obtained for ZDT3 test function.

**Fig. 18:**
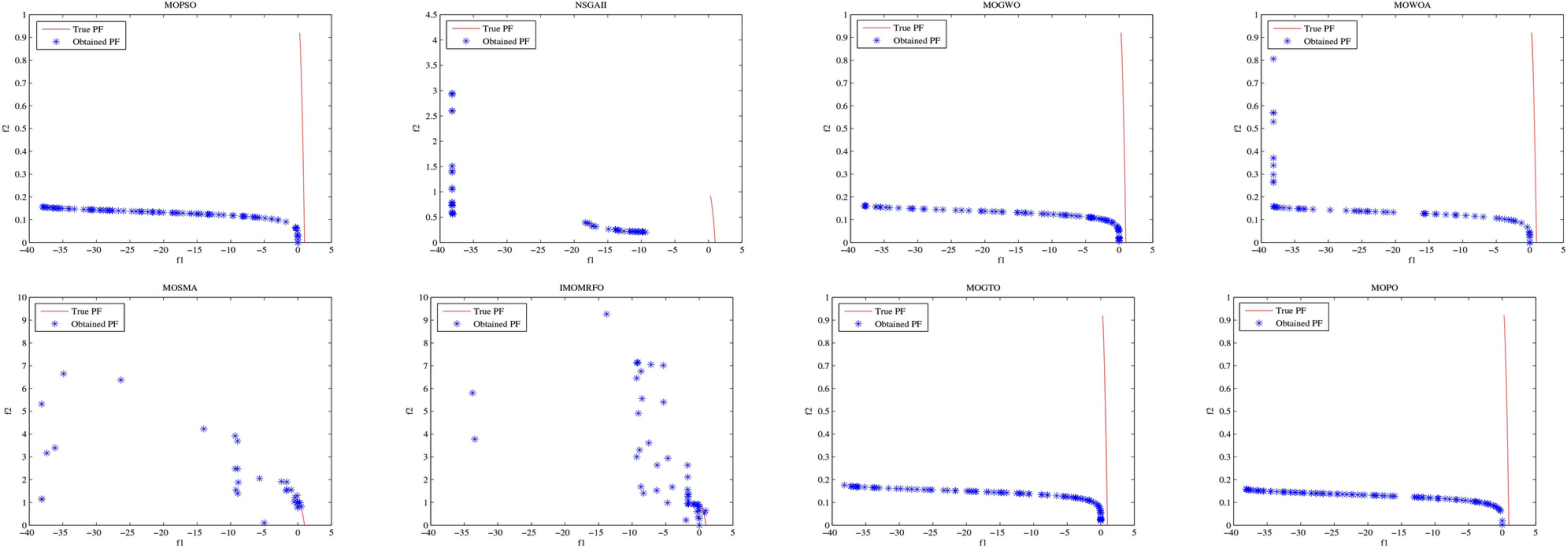
Pareto front acquired for ZDT6 function.

To validate the developed MOPO, four well-known ZDT test routines from^116^ were employed for comparison against other competing algorithms. The simulations were conducted over 20 independent runs. As shown in Table 6, the GD, Spacing, and Spread metrics were used to assess convergence and efficacy. The following deductions can be made in light of the results:

- Table 6 illustrates that, according to the GD measure that is utilized to validate the convergence output of simulation methods, the developed MOPO acquires superior findings over ZDT1, ZDT2, ZDT3, and ZDT6. Regarding spacing measure, the developed MOPO acquires the superior finding over ZDT1, ZDT2, and ZDT3 and is very competitive with MOPSO over ZDT6. For spread measure, the developed MOPO acquires the superior finding over ZDT1 compared to other rivals, whereas MOGTO achieves the superior finding over ZDT2 and ZDT3. Equally, the MOPSO acquires superior outcomes over ZDT6. Moreover, Figs. 15, 16, 17 and 18 depicts the coverage and convergence gained with each MO algorithm over all the considered ZDT routines.
- The performance of the comparison approaches is validated in Table 7 over four constrained benchmark routines, namely the OSY, BNH, KITA, and CONSTR test routines. The simulation was run 20 times independently. In addition, measures such as GD, spacing, and spread are calculated to demonstrate the effectiveness and convergence of comparing approaches. The resulting GD findings represent the better convergence of the developed MOPO over the restricted routines, as seen by the bolded findings in Table 7, which illustrate that the developed MOPO acquires the premium findings across all test cases in terms of GD measure. Table 7’s third and fourth columns guarantee that the created MOPO is resistant against competing solutions regarding spacing and spread measures. Furthermore, findings demonstrate a better diversity of the developed MOPO than all the test routines.

**Table 6:** GD, Spacing, and Spread metrics for algorithms on ZDT1, ZDT2, ZDT3, and ZDT6 test functions.

Algorithm		GD		Spacing		Spread	
		Mean	SD	Mean	SD	Mean	SD
ZDT1	MOPSO	0.0169	0.0009	0.0102	0.0022	0.7864	0.0384
	NSGA-II	0.0362	0.0012	0.0111	0.0010	0.6468	0.0340
	MOGWO	0.0227	0.0020	0.0240	0.0066	1.0308	0.0864
	MOWOA	0.0170	0.0061	0.0367	0.0162	1.2024	0.0934
	MOSMA	0.0199	0.0023	0.0134	0.0032	0.7989	0.1624
	IMOMRFO	0.0210	0.0019	0.0158	0.0036	0.7948	0.0591
	MOGTO	0.0207	0.0004	0.0110	0.0011	0.6716	0.0534
	MOPO	**0.0155**	**0.0004**	**0.0074**	**0.0006**	**0.5858**	**0.0496**
ZDT2	MOPSO	0.0127	0.0010	0.0118	0.0018	1.0182	0.0384
	NSGA-II	0.0411	0.0016	0.0095	0.0011	0.8390	0.0390
	MOGWO	0.0014	0.0005	0.0252	0.0070	1.0405	0.0748
	MOWOA	0.0009	0.0018	0.0377	0.0134	1.2256	0.1401
	MOSMA	0.0008	0.0003	0.0141	0.0013	0.6289	0.1016
	IMOMRFO	0.0157	0.0039	0.0246	0.0092	1.0168	0.0592
	MOGTO	0.0001	0.0001	0.0122	0.0013	**0.4235**	**0.0443**
	MOPO	**0.0001**	**0.0001**	**0.0086**	**0.0009**	0.5597	0.0938
ZDT3	MOPSO	0.1471	0.0095	0.0540	0.0193	0.8124	0.0291
	NSGA-II	0.2749	0.0221	0.0243	0.0036	0.8045	0.0367
	MOGWO	0.1554	0.0890	0.3332	0.1904	1.0405	0.2072
	MOWOA	0.2746	0.2628	0.1685	0.0880	0.8551	0.1449
	MOSMA	0.0076	0.0023	0.0429	0.0087	1.0815	0.1540
	IMOMRFO	0.7389	0.1966	0.2266	0.1359	0.8848	0.0626
	MOGTO	0.0005	0.0004	0.0389	0.0365	**0.7421**	**0.2700**
	MOPO	**0.0002**	**0.0000**	**0.0132**	**0.0006**	0.7550	0.0972
ZDT6	MOPSO	5.5101	4.6479	**0.2827**	**0.3404**	**0.9385**	**0.1266**
	NSGA-II	4.1147	0.8008	0.5229	0.4140	1.2641	0.1916
	MOGWO	4.3840	3.2942	0.8561	0.6238	1.1644	0.2132
	MOWOA	4.2619	2.4773	0.7543	0.8167	1.3316	0.2093
	MOSMA	2.4718	0.8532	1.9221	0.7057	1.3349	0.3132
	IMOMRFO	2.1858	1.3401	1.8867	1.8246	1.4996	0.3513
	MOGTO	3.3656	0.4054	2.3782	1.2591	1.6145	0.1913
	MOPO	**1.7742**	**1.0307**	0.3543	0.4001	1.2542	0.2332

Significant values are in (bold).

**Table 7:** GD, Spacing, and Spread metrics for algorithms on constrained test functions.

Algorithm		GD		Spacing		Spread	
		Mean	SD	Mean	SD	Mean	SD
OSY	MOPSO	2.4084	0.0003	0.0102	0.0011	0.0202	0.0021
	NSGA-II	2.6377	0.0003	0.0130	0.0013	0.0230	0.0014
	MOGWO	2.9519	0.0005	0.0272	0.0102	0.0372	0.0103
	MOWOA	2.4770	0.2400	0.0324	0.0225	0.0424	0.0226
	MOSMA	3.4038	0.0018	0.0292	0.0185	0.0392	0.0195
	IMOMRFO	2.9504	0.0006	0.0159	0.0029	0.0259	0.0039
	MOGTO	3.4043	0.0004	0.0229	0.0023	0.0329	0.0043
	MOPO	**2.0854**	**0.0002**	**0.0081**	**0.0008**	**0.0092**	**0.0007**
BNH	MOPSO	5.6992	0.0220	0.1167	0.0118	NaN	NaN
	NSGA-II	10.3070	0.1291	0.3404	0.0803	NaN	NaN
	MOGWO	10.2238	0.2251	0.6237	0.2284	NaN	NaN
	MOWOA	10.4558	0.4071	0.6356	0.1780	NaN	NaN
	MOSMA	10.5175	0.2317	0.4051	0.1110	NaN	NaN
	IMOMRFO	10.3911	0.1541	0.4427	0.1372	NaN	NaN
	MOGTO	10.3298	0.0770	0.3685	0.0431	NaN	NaN
	MOPO	**5.6350**	**0.0245**	**0.0994**	**0.0078**	NaN	NaN
KITA	MOPSO	2.4083	0.0002	0.0099	0.0012	0.0088	0.0036
	NSGA-II	2.6376	0.0005	0.0129	0.0012	0.0118	0.0029
	MOGWO	2.9515	0.0005	0.0252	0.0119	0.0241	0.0227
	MOWOA	3.2095	0.9370	0.0519	0.0343	0.0317	0.0254
	MOSMA	3.4045	0.0008	0.0285	0.0045	0.0174	0.0056
	IMOMRFO	2.9580	0.0242	0.0162	0.0022	0.0151	0.0044
	MOGTO	3.4045	0.0004	0.0221	0.0022	0.0131	0.0033
	MOPO	**2.0854**	**0.0001**	**0.0091**	**0.0008**	**0.0061**	**0.0009**
CONSTR	MOPSO	1.2998	0.0077	0.0375	0.0100	NaN	NaN
	NSGA-II	1.3875	0.0544	0.0451	0.0111	NaN	NaN
	MOGWO	1.5470	0.0403	0.1297	0.0551	NaN	NaN
	MOWOA	1.7358	0.7258	0.3481	0.3444	NaN	NaN
	MOSMA	2.0759	0.1197	0.4024	0.1500	NaN	NaN
	IMOMRFO	2.2365	0.1208	0.1336	0.0520	NaN	NaN
	MOGTO	1.8338	0.0084	0.0826	0.0060	NaN	NaN
	MOPO	**1.1249**	**0.0071**	**0.0371**	**0.0018**	NaN	NaN

###  Series 3: analysis of MO engineering problems

Engineering design challenges are crucial for assessing how well MAs operate. The performance of the proposed MOPO was evaluated in this study using four MO engineering design challenges: the speed reducer, disc brake, I-beam, and cantilever beam. Every MO algorithm is run 20 times in isolation. Additionally, the spacing metric is applied to the premium PFs produced for every MO algorithm; the given findings show how effective MOPO is at locating near-optimal PSs.

#### Speed reducer design

By addressing the speed reducer design optimization problem^117^, addresses the MO engineering design challenge. Seven design variables are included in this challenge: $X = [x1, x2, x3, x4, x5, x6, x7]^{T}$, which stands for the gear face width, teeth module, number of pinion teeth, distance between bearings 1, the distance between bearings 2, diameter of shaft 1, and diameter of shaft 2, in that order. As stated in Eq. 22, the speed reducer design problem is a multi-criteria optimization task with two weight and stress reduction goals that are meant to be minimized.

**Fig. 19:**
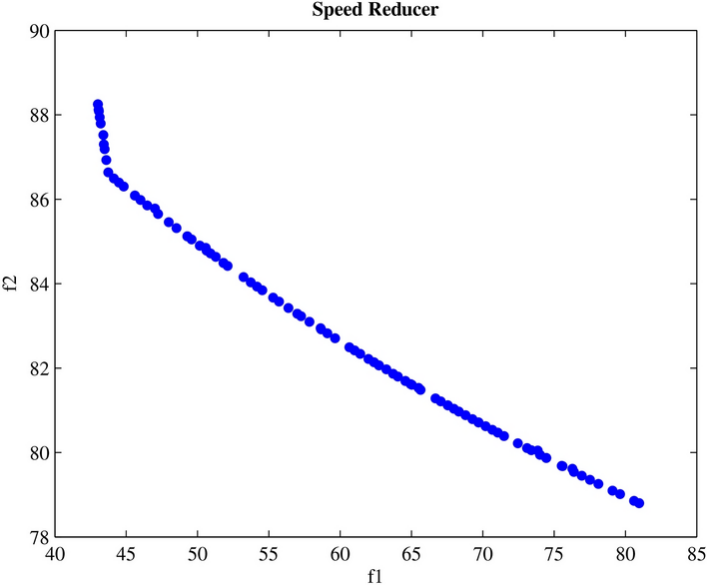
PF acquired by the developed MOPO for speed reducer design challenge.

Table 8 presents the best scores of the spacing metric for each MO method, while Fig. 19 shows the premium PFs acquired by the developed MOPO for the MO speed reducer design challenge.

22 $$\begin{aligned} & Minimize \left\{ \begin{array}{l} f_{\text{ weight } }=f_{1}(x)= \\ 0.7854 x_{1} x_{2}^{2}\left( 10 x_{3}^{2} / 3+14.933 x_{3}-43.0934\right) \\ -1.508 x_{1}\left( x_{6}^{2}+x_{7}^{2}\right) +7.477\left( x_{6}^{3}+x_{7}^{3}\right) \\ +0.7854\left( x_{4} x_{6}^{2}+x_{5} x_{7}^{2}\right) \\ f_{\text{ stress } }=f_{2}(\varvec{x})=\frac{\sqrt{\left( \frac{745 x_{4}}{x_{2} x_{3}}\right) ^{2}}+1.69 \times 10^{7}}{0.1 x_{6}^{3}} \end{array}\right. \end{aligned}$$

23 $$\begin{aligned} & \quad \text {Subject to }\left\{ \begin{array}{l} g_{1}(\varvec{x})=1 / 27-1 /\left( x_{1} x_{2}^{2} x_{3}\right) \ge 0 \\ g_{2}(\varvec{x})=1 / 397.5-1 /\left( x_{1} x_{2}^{2} x_{3}^{2}\right) \ge 0 \\ g_{3}(\varvec{x})=1 / 1.93-x_{4}^{3} /\left( x_{2} x_{3} x_{6}^{4}\right) \ge 0 \\ g_{4}(\varvec{x})=1 / 1.93-x_{5}^{3} /\left( x_{2} x_{3} x_{7}^{4}\right) \ge 0 \\ g_{5}(\varvec{x})=40-x_{2} x-3 \ge 0 \\ g_{6}(\varvec{x})=12-x_{1} / x_{2} \ge 0 \\ g_{7}(\varvec{x})=x_{1} / x_{2}-5 \ge 0 \\ g_{8}(\varvec{x})=x_{4}-1.5 x_{6}-1.9 \ge 0 \\ g_{9}(\varvec{x})=x_{5}-1.1 x_{7}-1.9 \ge 0 \\ g_{10}(\varvec{x})=1,300-f_{2}(\varvec{x}) \ge 0 \\ g_{11}(\varvec{x})=1,100-\frac{\sqrt{\left( \frac{745 x_{5}}{x_{2} x_{3}}\right) ^{2}}+1.275 \times 10^{8}}{0.1 x_{7}^{3}} \ge 0 \end{array}\right. \end{aligned}$$

where the upper and lower boundaries of the design variables are represented in the following:

$$\begin{array}{l} 2.6 \le x_{1} \le 3.6, 0.7 \le x_{2} \le 0.8, 17 \le x_{3} \le 28, 7.3 \le x_{4} \le 8.3, \\ 7.3 \le x_{5} \le 8.3, 2.9 \le x_{6} \le 3.9, 5.0 \le x_{7} \le 5.5. \end{array}$$

**Table 8:** The findings of the MO methods utilizing spacing measure over speed reducer design challenge.

Problem	Method	Spacing	
		Mean	SD
Speed	MOPSO	0.3541	0.0456
	NSGA-II	0.3906	0.0351
	MOGWO	0.8201	0.2643
	MOWOA	0.9918	0.4794
	MOSMA	0.5841	0.0709
	IMOMRFO	0.4261	0.0780
	MOGTO	0.4854	0.0569
	MOPO	**0.2267**	**0.0187**

#### Disc brake design

**Fig. 20:**
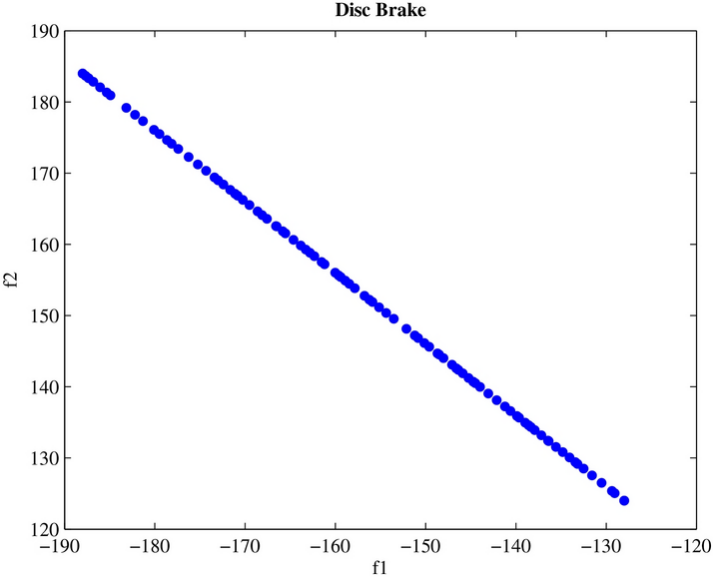
PF acquired by the developed MOPO for disc brake design challenge.

Optimizing the disc brake design is the second MOO task tackled by^118^. The aim is to find the ideal dimension scores *X* for the design. $X = [x1, x2, x3, x4]^{T}$ is the equation containing the four design variables. The discs’ inner and outer radii are denoted by *x*1 and *x*2, their engagement force by *x*3, and their number of friction surfaces by *x*4. As seen in Eq. 24, the disc brake design issue is an MOP with two minimization objectives: minimizing the brake’s mass (*f*1) and stopping time (*f*2). Table 9 displays the spacing measure premium scores for every MO approach. Furthermore, the premium gained PFs for the produced MOPO in the disc brake design challenge are visualized in Fig. 20.

24 $$\begin{aligned} Minimize \left\{ \begin{array}{l}f_{1}(\varvec{x})=4.9 \times 10^{-5}\left( x_{2}^{2}-x_{1}^{2}\right) \left( x_{4}-1\right) \\ f_{2}(\varvec{x})=\frac{9.82 \times 10^{6}\left( x_{2}^{2}-x_{1}^{2}\right) }{x_{3} x_{4}\left( x_{2}^{3}-x_{1}^{3}\right) }\end{array}\right. \end{aligned}$$

25 $$\begin{aligned} \text {Subject to} \left\{ \begin{array}{l}g_{1}(\varvec{x})=\left( x_{2}-x_{1}\right) -20 \ge 0 \\ g_{2}(\varvec{x})=30-2.5\left( x_{4}+1\right) \ge 0 \\ g_{3}(\varvec{x})=0.4-x_{3} /\left( 3.14\left( x_{2}^{2}-x_{1}^{2}\right) \right) \ge 0 \\ g_{4}(\varvec{x})=1-\frac{2.22 \times 10^{-3} x_{3}\left( x_{2}^{3}-x_{1}^{3}\right) }{\left( x_{2}^{2}-x_{1}^{2}\right) ^{2}} \ge 0 \\ g_{5}(\varvec{x})=\frac{ \left. 2.66 \times 10^{-2} x_{3} x_{4}^{3}-x_{1}^{3}\right) }{\left( x_{2}^{2}-x_{1}^{2}\right) }-900 \ge 0\end{array}\right. \end{aligned}$$

where $$55 \le x_{1} \le 80,75 \le x_{2} \le 110,1,000 \le x_{3} \le$$ 3,000 , and $$2 \le x_{4} \le 20$$.

**Table 9:** The findings of the MO methods utilizing spacing measure over disc brake design challenge.

Problem	Algorithm	Spacing	
		Mean	SD
Disc brake	MOPSO	0.5506	0.0546
	NSGA-II	0.4683	0.0398
	MOGWO	0.6389	0.1288
	MOWOA	1.2166	0.4102
	MOSMA	0.5610	0.0314
	IMOMRFO	0.7114	0.1610
	MOGTO	0.4485	0.0358
	MOPO	**0.3917**	**0.0201**

Significant values are in (bold).

#### I-beam design challenge

**Fig. 21:**
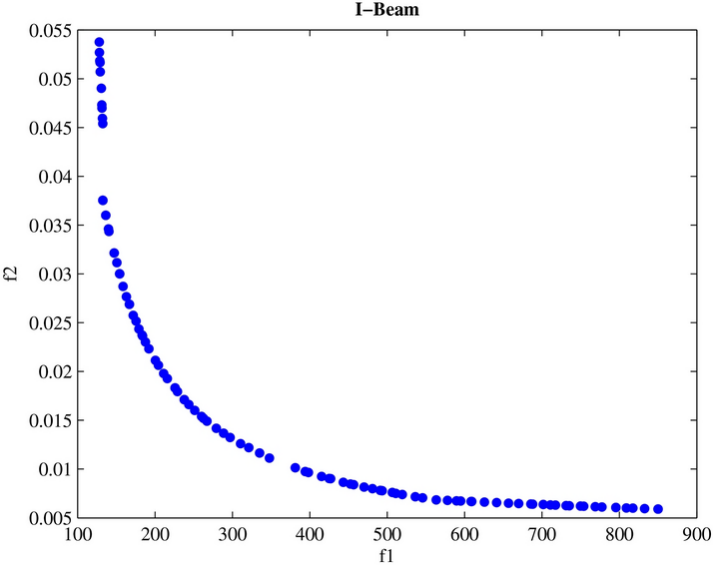
PF acquired by the developed MOPO for I-Beam design challenge.

The I-Beam design challenge focuses on determining the beam’s optimal dimension scores *X*, represented as $X = [x1, x2, x3, x4]^{T}$. This is a multi-criteria optimization problem with two minimization objectives, as described in Eq. 26: minimizing the cross-sectional area (*f*1) and minimizing the deflection of the beam (*f*2)^119^. Table 10 presents the premium scores of the spacing measure for each MO method, while Fig. 21 illustrates the premium acquired PFs for the developed MOPO in the I-beam design challenge.

26 $$\begin{aligned} Minimize \left\{ \begin{array}{c} f_{1}(X)=2 x_{2} x_{4}+x_{3}\left( x_{1}-2 x_{4}\right) \\ f_{2}(X)=\frac{60000}{x_{3}\left( x_{1}-2 x_{4}\right) ^{3}+2 x_{2} x_{4}\left[ 4 x_{4}^{2}+3 x_{1}\left( x_{1}-2 x_{4}\right) \right] } \end{array}\right. \end{aligned}$$

where constraints of problem dimensions are $10 \le x_{1} \le 80,10 \le x_{2} \le 50, \quad 0.9 \le x_{3}$ and $x_{4} \le 5$. The design of the MO I-beam problem is under the following restriction:

$$\text {Subject to: }g_{1}(X)=\frac{180000 x_{1}}{x_{3}\left( x_{1}-2 x_{4}\right) ^{3}+2 x_{2} x_{4}\left[ 4 x_{4}^{2}+3 x_{1}\left( x_{1}-2 x_{4}\right) \right] } +\frac{15000 x_{2}}{\left( x_{1}-2 x_{4}\right) x_{3}^{3}+2 x_{4} x_{2}^{3}} \le 16.$$

**Table 10:** The findings of the MO methods utilizing spacing measure over I-Beam design challenge.

Issue	Method	Spacing	
		Mean	SD
I-Beam	MOPSO	6.3123	1.6793
	NSGA-II	4.4707	0.6720
	MOGWO	6.0336	0.8313
	MOWOA	9.6671	2.5451
	MOSMA	9.5571	2.2012
	IMOMRFO	6.9923	2.8766
	MOGTO	4.0309	0.3819
	MOPO	**3.3852**	**0.3121**

Significant values are in (bold).

#### Cantilever beam design

**Fig. 22:**
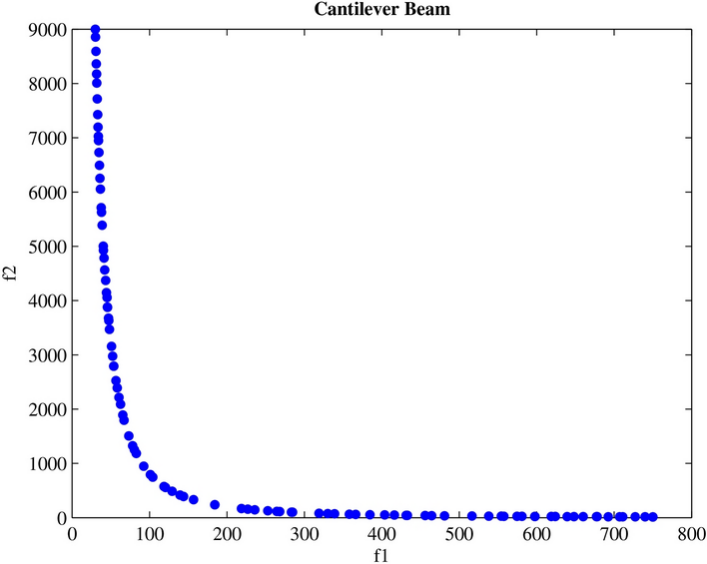
PF acquired by the developed MOPO for cantilever beam design challenge.

The objective of the cantilever beam design problem is to determine the optimal dimension scores *Q* for the beam, represented as $Q = [d, l]^{T}$, where *d* denotes the beam’s diameter and *l* represents the length of the beam. This is a multi-criteria optimization problem with two minimization objectives, as outlined in Eq. 27: minimizing the weight (*f*1) and minimizing the deflection of the beam (*f*2)^120^. Table 11 presents the premium scores of the spacing measure for each MO method. In contrast, Fig. 22 illustrates the premium acquired PFs for the developed MOPO in the cantilever beam design challenge.

27 $$\begin{aligned} Minimize \left\{ \begin{array}{l} f_{1}(\varvec{Q})= \text{ weight } =\frac{\rho \pi d^{2} l}{4} \\ f_{2}(\varvec{Q})= \text{ deflection } =\frac{64 p l^{3}}{3 E \pi d^{4}} \end{array}\right. \end{aligned}$$

The constraints for the problem dimensions are $10 \le d \le 50$ and $200 \le l \le 1000$. The following constraints apply to the cantilever beam design problem:

28 $$\begin{aligned} \text {S. t. }\left\{ \begin{array}{l} g_{1} = \sigma \le \text{S}_{\text{y}} \text{ i.e., } \frac{32 p l}{\pi d^{3}} \le 300 \\ g_{2} = \delta \le \delta _{\max } \text{ i.e., } \frac{64 p l^{3}}{3 E \pi d^{4}} \le 5 \end{array}\right. \end{aligned}$$

where $\text{S}_{\text{y}} = 300$ Mpa and $\delta _{\max } = 5 \;$ mm.

**Table 11:** The findings of the MO methods utilizing spacing measure over cantilever beam design challenge.

Problem	Algorithm	Spacing	
		Mean	SD
Cantilever	MOPSO	83.1777	15.3354
	NSGA-II	64.5646	5.0963
	MOGWO	122.1235	26.3610
	MOWOA	201.9251	104.5783
	MOSMA	69.9611	5.3349
	IMOMRFO	100.0731	29.9990
	MOGTO	64.9519	4.5985
	MOPO	**61.8349**	**4.2252**

Significant values are in (bold).

###  Series 4: analysis of real-world MO helical coil spring problem for automotive application

The helical coil spring is manufactured of IS4454 and has a constant axial compressive load. The primary goal is to develop a coil spring for use in a commercial three-wheeler automobile utilizing the MOO approach^121,122^. The design of a helical spring is a multi-criteria optimization issue with two goals: (i) maximize strain energy and (ii) minimize spring volume. Furthermore, the design process is subject to a set of eight limitations in order to achieve the required criteria. The spring has three design factors (Eq. (29)): mean coil diameter (*D*), wire diameter (*d*), and number of active coils (*Na*). The parameters used in the helical coil spring design problem are listed in Table 12. Moreover, the optimization findings in Table 13 show that the proposed MOPO algorithm produces more reliable solutions with less volume spring area and more durable strain energy than the other competitors; additionally, the design variables of spring designs generated by the proposed MOPO are near-optimal and could be utilized in automotive applications. Based on their priorities, decision-makers can choose the best spring design from the Pareto solutions offered by the proposed MOPO for the automobile industry.

29 $$\begin{aligned} \text{ DesignVector, } \vec {X}=[d, D, N a]^T \end{aligned}$$

Where *d* indicates wire diameter, *D* indicates coil diameter, and *Na* indicates number of coil turns. The spring volume (minimization goal) is depicted in Eq. (30), while the strain-energy (maximization goal) is illustrated in Eq. (31) as follows:

30 $$\begin{aligned} f_1(X)= \text{ crosssectionalarea } \times \text{ springlength } =\frac{\pi }{4} D d(N a+2) \end{aligned}$$

31 $$\begin{aligned} f_2(X)= \text{ strain } - \text{ energy } =\frac{\pi }{4} f_{\max } \delta \end{aligned}$$

Where, $f_{\max }$ indicates maximum load while $\delta$ denotes deflection of the spring in Eq. (32) as follows:

32 $$\begin{aligned} \delta =\frac{8 F D^3 N}{G d^4} \end{aligned}$$

The design process subjects to eight constraints as follows:

33 $$\begin{aligned} \left\{ \begin{array}{l} g_1(X), \text{ ShearStress } =\frac{8 C K F_{\max }}{\pi ^2}-S \le 0 \\ g_2(X), \text{ Springlengthunderloading } =l-l_{\max } \le 0 \\ g_3(X), \text{ Springlengthunderloading } =d_{\min }-d \le 0 \\ g_4(X), \text{ CoilDiameter } =(D-d)-d_{\max } \le 0 \\ g_5(X), \text{ Prelod } - \text{ deflection } =\delta _p-\delta _{p m} \le 0 \\ g_6(X), \text{ Max.deflectionLoading } =\delta _p \frac{\left( F_{\max }-F_p\right) }{k} \\ \quad +1.05(N+2) D-l \le 0 \\ g_7(X), \text{ Pre } - \text{ loadandMax.Loadingdeflection } =\delta _w \frac{\left( F_{\max }-F_p\right) }{k} \le 0 \\ g_8(X), \text{ Springindex } =6.3-C \le 0 \end{array}\right. \end{aligned}$$

**Table 12:** Parameters utilized in the helical coil spring design challenge.

Parameter name	Abbreviation	Value
Max. load	$F_{max}$	3200 N
Max. shear-stress (allowable)	*S*	1303 MPa
Max. free length	$l_{max}$	315 mm
Min. wire-diameter	$d_{min}$	10 mm
Max. outside spring diameter	$d_{max}$	88 mm
Compression force (preload)	$F_p$	1334 N
Max. allowable deflection	$\delta _{pm}$	152 mm
Deflection	$\delta _{w}$	32 mm
Shear-modulus	*G*	81370 MPa

**Table 13:** The findings of the MO methods over helical coil spring challenge for automotive application.

Method	Challenge design objectives		Challenge design parameters		
	Volume(mm^3^)	Strain energy	d (mm)	D (mm)	Na
MOPSO	4.11E+07	2.27E+09	2.994	20.0	10.50
	4.39E+07	2.19E+09	2.966	20.0	10.50
	4.68E+07	2.12E+09	2.940	20.0	10.50
NSGA-II	1.50E+07	1.61E+10	4.302	20.7	16.00
	1.50E+07	1.60E+10	4.273	20.9	16.00
	1.51E+07	1.56E+10	4.244	20.9	15.95
MOGWO	2.24E+07	3.33E+09	3.306	20.0	10.50
	2.62E+07	2.97E+09	3.210	20.0	10.50
	2.88E+07	2.78E+09	3.159	20.0	10.50
MOWOA	1.48E+07	1.30E+10	4.192	20.0	16.00
	1.48E+07	1.24E+10	4.143	20.0	16.00
	1.49E+07	1.23E+10	4.136	20.0	15.98
MOSMA	1.50E+07	1.16E+10	4.156	20.0	15.99
	1.48E+07	1.25E+10	4.147	20.0	15.98
	1.50E+07	1.16E+10	4.100	20.0	15.55
IMOMRFO	1.50E+07	1.16E+10	4.089	20.0	15.69
	1.56E+07	8.82E+09	3.939	20.0	13.87
	1.59E+07	7.60E+09	3.849	20.0	13.11
MOGTO	2.24E+07	3.33E+09	3.306	20.0	10.50
	2.62E+07	2.97E+09	3.210	20.0	10.50
	2.88E+07	2.78E+09	3.159	20.0	10.50
MOPO	1.48E+07	1.25E+10	4.145	20.0	16.00
	1.49E+07	1.22E+10	4.124	20.0	16.00
	1.53E+07	1.11E+10	4.030	20.0	16.00

### Observations from the experiments

In summary, the following observations from the experiments are:

1. The proposed MOPO, being an optimization method, presents certain advantages:
  The experimental findings showed that the MOPO has great coverage and convergence abilities (see Tables 2, 3, and 4). The MOPO algorithm inherits PO’s high convergence, whilst the archive and non-dominated sorting maintenance methods contribute to its high coverage.
  The main mechanisms that guarantee convergence in PO and MOPO are the diversity of four distinct attitudes (foraging, remaining, communicating, and fear of strangers) and the use of Levy distribution.
  Furthermore, a comparison is made between the archive solutions and the NDSs. This method highlights the coverage rate of the Pareto optimal front solutions (see Fig. 12) since solutions in the intensity zones in the archive are more likely to be discarded.
2. The proposed MOPO demonstrates superior performance and effective convergence towards the true Pareto optimal sets compared to other MO algorithms evaluated. However, the MOPO has some limitations, as outlined below:
  Based on the PO, MOPO incurs relatively high computational costs. This is due to PO’s requirement to mathematically simulate the behavior of the Pyrrhura Molinae parrot, including foraging, remaining, communicating, and responding to threats, which necessitates complex calculations at each cycle with time complexity of up to $O(S_{in}^2 \cdot \log (S_{in}))$.
  MOPO may occasionally become trapped in local optima, as indicated by the convergence technique used in PO to accelerate convergence (see Table 3 for MMF14_a, MMF11_I, and MMF13_I).
  For MOPs with two or three objectives, the MOPO method is primarily intended. When the number of objectives rises, it becomes less effective, and a greater percentage of the solutions become non-dominated, quickly filling the archive. As a result, MOPO works well on issues with less than four objectives.
  Furthermore, MOPO is specifically tailored for continuous variable optimization problems, as the PO algorithm is designed to address such problems exclusively.
3. The similarities and differences of the proposed MOPO with the contemporary MO algorithms are as follows:
  The proposed MOPO is similar to previous Pareto dominance-based algorithms, such as the NSGA-II, in that it uses the non-dominated sorting mechanism to get the Pareto solution for the population.
  The proposed MOPO uses the archive memory to preserve the NDSs discovered throughout the optimization process, just like other archive-based multi-objective algorithms.
  The proposed MOPO uses the original PO updating method to investigate additional NDSs with high convergence and coverage rates, which improves the MOPO performance over previous similar multi-objective algorithms.

## Limitations and advantages of the proposed MOPO

This section specifies some of the advantages and weaknesses of the proposed MOPO method. The advantages listed below demonstrated how effective the MOPO algorithm was at resolving different challenging optimization issues:

- The proposed MOPO demonstrates strong convergence and coverage abilities, leveraging the diverse behavioral strategies (foraging, remaining, communicating, and fear of strangers) inspired by the Parrot Optimizer (PO). This contributes to maintaining a balanced exploration-exploitation trade-off.
- The integration of an outward archive and non-dominated sorting improves the maintenance of Pareto optimal solutions, enhancing performance metrics such as PSP, HV, and IGDX.
- MOPO provides robust performance across a diverse set of constrained and unconstrained engineering design challenges, as shown in Tables 2, 3 and 4.

The proposed MOPO demonstrates strong performance across various benchmarks and real-world problems. However, like all algorithms, it is subject to limitations and areas where future research can contribute to further advancements. Some key limitations and opportunities for improvement include:

- Most current algorithms, including MOPO, are tailored for a specific dimensionality of objectives. Designing a unified framework capable of effectively handling a dynamic range of objectives remains an open research challenge.
- Integrating decision-maker preferences into the evolutionary process enhances practical applicability. Future extensions of MOPO could incorporate interactive mechanisms to adaptively refine solutions based on user-defined trade-offs.
- Like other MOEAs, MOPO requires careful parameter tuning, which can be computationally expensive. Developing adaptive parameter control strategies to improve robustness and reduce computational costs is a critical direction for improvement.

## Conclusion and future directions

This paper introduced MOPO, a multi-objective optimization algorithm inspired by the Parrot Optimizer (PO). MOPO extends PO’s capabilities by incorporating an external archive and utilizing Pareto dominance theory to refine its optimization strategy. We benchmarked MOPO against seven leading multi-objective optimization algorithms, such as MOPSO, NSGA-II, MOGWO, MOWOA, MOSMA, IMOMRFO, and MOGTO. The evaluation leveraged the CEC’2020 benchmark function, which contains 24 test functions with diverse PF and set characteristics. MOPO’s performance was rigorously assessed using multiple indicators such as Pareto Sets Proximity (PSP), Inverted Generational Distance in Decision Space (IGDX), and Hypervolume (HV). Additionally, MOPO’s effectiveness was demonstrated through extensive testing on eight popular constrained and unconstrained test issues and four engineering design issues using metrics like Generational Distance (GD), spacing, and maximum spread. Moreover, the real-world multi-objective optimization of helical coil spring for automotive application was tackled to depict the reliability of the proposed MOPO to solve real-world challenges. Our findings reveal that MOPO generates high-quality solutions and closely approaches the optimal PF. The algorithm’s power to retain the best candidates among true Pareto optimal solutions is enhanced by integrating a CD operator and an archive of NDS. This design ensures both effective exploration and exploitation throughout the optimization process.

Looking ahead, MOPO shows significant potential for application in machine learning domains, such as feature selection, parameter optimization, and data preprocessing. Future work will further refine MOPO and explore its significance in solving additional complex real-world issues.

## Data Availability

The data sets provided during the current study are available: In^116^ and https://www3.ntu.edu.sg/home/epnsugan/index_files/CEC2020/CEC2020-3.htm.

## Abbreviations

PO: Parrot optimizer
MOPO: Multi-objective parrot optimizer
IMOMRFO: Improved multi-objective manta-ray foraging optimization
MOGTO: Multi-objective gorilla troops optimizer
MOGWO: Multi-objective grey wolf optimizer
MOWOA: Multi-objective whale optimization algorithm
MOSMA: Multi-objective slime mold algorithm
MOPSO: Multi-objective particle swarm optimization
NSGA-II: Non-dominated sorting genetic algorithm II
MOEA: Multi-objective evolutionary algorithm
CEC: Congress on evolutionary computation
PSP: Pareto set proximity
IGDX: Inverted generational distance
HV: Hypervolume
GD: Generational distance
MH: Metaheuristic
MO: Multi-objective
MOP: Multi-objective problem
MOO: Multi-objective optimization
PF: Pareto front
PS: Pareto set
NDS: Non-dominated solution
NDR: Non-dominated ranking
CD: Crowding distance
